# Cyclic microchip assay for measurement of hundreds of functional proteins in single neurons

**DOI:** 10.1038/s41467-022-31336-x

**Published:** 2022-06-21

**Authors:** Liwei Yang, Avery Ball, Jesse Liu, Tanya Jain, Yue-Ming Li, Firoz Akhter, Donghui Zhu, Jun Wang

**Affiliations:** 1 Multiplex Biotechnology Laboratory, Department of Biomedical Engineering, Stony Brook University, Stony Brook, NY 11794 USA; 2 Chemical Biology Program, Memorial Sloan Kettering Cancer Center, New York, NY USA; 3 Programs of Neurosciences, Weill Graduate School of Medical Sciences of Cornell University, New York, NY USA; 4 Programs of Pharmacology, Weill Graduate School of Medical Sciences of Cornell University, New York, NY USA; 5 Department of Biomedical Engineering, Stony Brook University, Stony Brook, NY 11794 USA

**Keywords:** Biotechnology, Biomedical engineering, Immunohistochemistry, Animal disease models, Alzheimer's disease

## Abstract

Despite the fact that proteins carry out nearly all cellular functions and mark the differences of cells, the existing single-cell tools can only analyze dozens of proteins, a scale far from full characterization of cells and tissue yet. Herein, we present a single-cell cyclic multiplex in situ tagging (CycMIST) technology that affords the comprehensive functional proteome profiling of single cells. We demonstrate the technology by detecting 182 proteins that include surface markers, neuron function proteins, neurodegeneration markers, signaling pathway proteins, and transcription factors. Further studies on cells derived from the 5XFAD mice, an Alzheimer’s Disease (AD) model, validate the utility of our technology and reveal the deep heterogeneity of brain cells. Through comparison with control mouse cells, we have identified differentially expressed proteins in AD pathology. Our technology could offer new insights into cell machinery and thus may advance many fields including drug discovery, molecular diagnostics, and clinical studies.

## Abstract (web summary)

Current single-cell tools are limited by the number of proteins they can analyse. Here the authors report a single-cell cyclic multiplex in situ tagging (CycMIST) method for functional proteome profiling of single cells, allowing multiple rounds of multiplexing of the same single cells on a microchip.

## Introduction

Recent advancement of single-cell omics technologies has revolutionized the study of complex biological systems and has significantly impacted on precision medicine^1,2^. A scale at the omics level is necessary since a typical animal cell contains numerous proteins, transcripts and other molecules that participate in normal functions^3,4^. Propelled by the broadly available sequencing tools, single-cell transcriptomics has been frequently applied in many human diseases to reveal the genome-wide gene expression among individual cells^5,6^. Proteins, however, are not amplifiable like DNAs, and thus protein analysis in single cells has not reached the scale comparable to transcriptomes. Proteins largely represent cell functions and biomarkers for disease diagnosis and cell type classification^7^. Hundreds of known functional proteins represent cell identity, drug targets, clinical biomarkers, signaling networks, transcriptional factors, functional readouts of proliferation, cell cycle status, metabolism regulation, and apoptosis markers^7,8^. Single-cell functional proteomics is irreplaceable particularly for disease pathology and signal pathway analyses, since the correlation of gene expression and protein expression is generally ~0.4 and almost no correlation exists for low expression proteins (particularly for signaling proteins)^9–11^. Furthermore, protein-level diagnosis desirably offers more direct implication of pathogenesis and potential drug targets^8^.

A variety of multiplex strategies have been introduced to achieve the measurement of dozens of proteins per cell, but they are still far from functional proteome level^4,12^. While conventional fluorescence flow cytometry can detect up to 17 colors simultaneously^13^, it is barely used in practice due to high cost of instrumentation and reagents. Mass cytometry, a technique utilizing transition element isotopes as labelling tags instead of fluorophores, offers a higher multiplexity of ~40 s in single-cell analysis^14^. Similarly, multiplexed ion beam imaging by time of flight (MIBI-TOF) leverages isotope tagging in immunohistochemistry to achieve high-resolution imaging of 36 proteins for tumor specimens^15^. Another strategy is to reiteratively stain cells with fluorophore tagged antibodies for a few cycles to overcome the limitation of fluorescence spectrum overlapping^16–22^. The same cells are imaged in each cycle and are registered, so a multiplexity of 60s–80s is achievable^18^. This is a convenient, albeit laborious, technique that does not require special instruments. Nevertheless, for all the immunohistochemistry-based techniques, the high multiplexity is associated with the high risk of ‘parking’ issue due to limited room for binding of many antibodies to protein clusters^23,24^. A cell is packed with macromolecules, cytoskeleton and organelles, and therefore it cannot accommodate too many antibodies that are normally large molecules. These factors limit the practical use of multiplexed assays at less than 50 proteins per batch^25^, while many of them keep the spatial information without losing tissue integrity. Theoretically, the parking issue could be avoided by removing the antibodies and re-applying another batch of antibodies for labeling. It’s reported that around 20 cycles of staining are possible based on conventional immunofluorescence staining method^16^; however, it takes almost 2 weeks to achieve the measurement of 60 proteins/cell^18,26^. Thus, while the currently available high-multiplex protein detection methods can retain spatial information, the speed of processing and the maximum multiplexity are limited. The other emerging methods based on mass spectrometry and microchip western blotting can potentially overcome the bottleneck of multiplexity, but they are still not practically useful yet for routine high-multiplex assays in single cells^27,28^.

DNA barcoding on microarrays is a robust genome-wide assay that can analyze thousands of molecules simultaneously^29^, although it is not at the single-cell level yet. To transform its utility in protein assays, barcoded DNAs are tagged with antibodies where the antibodies recognize targets and DNAs confer signal^24,30,31^. A plethora of strategies can be adopted for signal amplification, enhancement and multiplexed detection with the aid of barcoded DNAs. The signal readout can rely on PCR^32^, fluorescence imaging^33,34^, NanoString^35^, and even next-generation sequencing^31^, while their multiplexity is still limited by antibody labeling. Miniaturized DNA barcode arrays enable the measurement of up to 35–40 proteins in single cells when the array is combined with a microfluidic chip for single-cell manipulation^36^. Further improvement of multiplexity is limited by the size of DNA arrays which are normally 20 μm for each^37^. In our previous work, we developed a multiplex in situ tagging (MIST) array technology on DNA encoded microbeads for single-cell cytokine assays^38,39^. The small size of microbeads holds great potential for functional proteomic assays in single cells since MIST has increased the assay capacity by 100 times.

Herein, we report a single-cell cyclic multiplexed in situ tagging (CycMIST) platform that can analyze hundreds of protein targets in single cells with high throughput and high sensitivity using a common imaging platform. The high protein content is achieved by the multi-round labeling and multi-cycle decoding process based on the CycMIST platform. Each single cell in a microchip microwell can be stained for 4 rounds by a cocktail of UV-cleavable DNA barcoded antibodies, and for each staining the DNA oligos can be released by UV irradiation and captured by a MIST array. The decoding process on a MIST array permits identification of up to 50 types of released DNAs at one time. Thus, the whole process overcomes the typical parking issue related with high multiplexity while the total multiplexity can increase by 40–50 for each round. We have thoroughly validated the technology and characterized its performance. As a proof-of-concept study, the CycMIST has been applied to differentiated mouse neuroblastoma Neuro-2a (N2a) cells for analysis of 182 proteins that include surface markers, neuron function proteins, neurodegeneration markers, signaling pathway proteins and transcription factors. An additional study on tissue from the mouse pre-frontal cortex found that CycMIST can profile the functional proteome of individual brain cells and distinguish molecular features between WT tissue and Alzheimer’s Disease (AD) tissue from a 5xFAD mouse model. The CycMIST technology should be broadly applicable to various complex diseases in the future for mechanistic studies at the single-cell functional proteomics level.

## Results

### Workflow of single-cell CycMIST technology

**Fig. 1:**
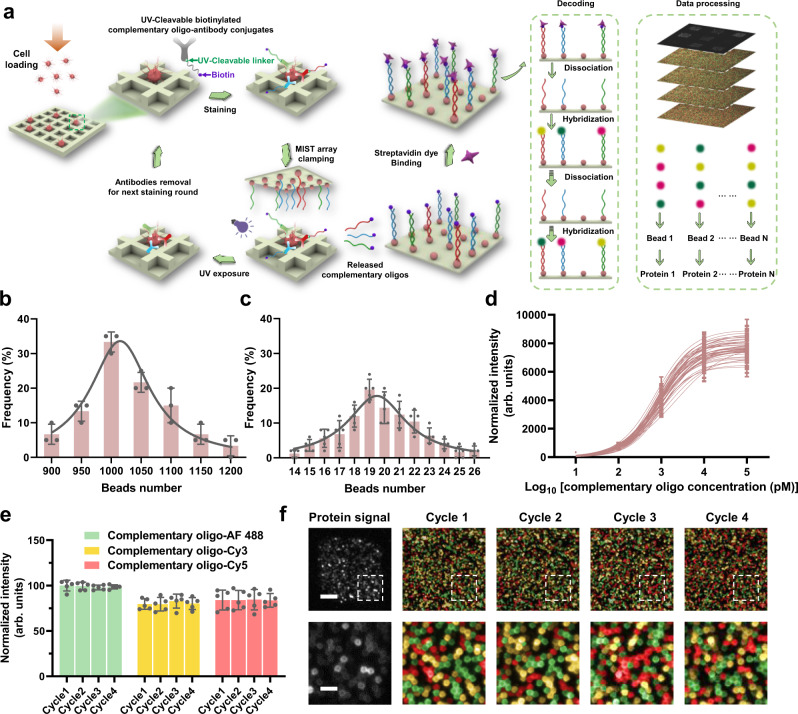
Overview of CycMIST technology for single-cell functional proteome analysis. **a** Schematic illustration of the CycMIST process to analyze multiple proteins through the MIST microbeads array. **b** Distribution of the number of oligo DNA-coated microbeads on each 75 μm × 75 μm area of a MIST array that is corresponding to a PDMS microwell, *n* = 3 independent MIST array. **c** Distribution of the number of same kind oligo DNA-coated microbeads on the same MIST array, *n* = 5 independent MIST array. **d** Characterization of the CycMIST sensitivity by varying the concentrations of 50 biotinylated complementary oligo DNAs on the MIST array, n = 10 independent experiments. This is the same procedure in single-cell protein detection experiments except cell loading and conjugate binding. **e** Consistency of fluorescence intensities for 4 decoding cycles and for 3 fluorescent color dyes (Alexa Fluor 488, Cy3 and Cy5), *n* = 5 independent experiments. **f** Sample images of multiplexed assay of 50 proteins from a single cell by CycMIST and the 4 decoding cycles images. The greyscale images are protein detection result, and the color images are the decoding cycles from cycle 1 to cycle 4. The bottom panel is the zoom-in images from the squares in the up panel. Scale bar: 20 μm (up panel); 5 μm (bottom panel). Data are presented as mean values ± SD of more than three independent experiments, and error bars are within symbol size if not shown. The term (arb. units) is abbreviated for arbitrary units.

The CycMIST platform involves multi-round staining/dissociation and multi-cycle decoding processes for multiplexed single-cells protein profiling (Fig. 1a). Each round can detect up to 50 proteins simultaneously for a single cell, and the decoding process for each round is to assign the protein ID to the detected protein signals. Single cells were attached on the surface of PDMS microwells that were sealed with part of the MIST array. The cells were stained with antibody conjugates that were tagged with UV cleavable oligo DNAs. Upon UV light exposure, the oligos were released and detected by the microbeads on the MIST array within the microwell area. Then the array was separated from the PDMS microwells for further decoding process, while the cells were retained in the PDMS microwells and were processed to remove the antibody conjugates to recover the antigens. The next round started with staining of another batch of antibody conjugates with UV cleavable oligo DNAs, and the same procedure was used as the first round. Thus, the total multiplexity in protein detection depends on the number of rounds and the number of proteins detected for each round.

Specifically, each antibody conjugate for cell staining contains two UV cleavable linkers between the complementary oligo DNA and the antibody, and one end of the complementary oligo has a biotin moiety. After UV cleavage, those bound antibodies on the cells are dissociated by a regeneration buffer, initiating the next staining round by another conjugates panel for different targeting. Each round of staining/dissociation generates a MIST array with fluorescence signals corresponding to protein abundance which is denoted as protein signal. Decoding on the MIST array requires multiple cycles which are dependent on the number of targets (multiplexity). It relies on the unique, consecutive color change on the same microbead to recognize the DNA sequence, or the detected protein, by this microbead, since each DNA sequence corresponds to only one type of protein target as is predesigned (Supplementary Data 1), and all DNAs are orthogonal to each to each other. To track the same microbead from protein detection to decoding experiments for all ~25 million microbeads of a MIST array, the images from protein signal and the images of decoding are well aligned by a Matlab program (Supplementary Software). Notably, the MIST array could be decoded beforehand or after protein detection. In the current report, we demonstrate the detection of 182 proteins through 4 rounds of staining/dissociation and 4 cycles of decoding process with 3 colors dye. Higher multiplexity can be simply achieved by more rounds of staining/dissociation with up to 50 proteins for each, and thus hundreds of proteins can be detected for single cells. Theoretically, an unprecedented level of multiplexity (K × M^N^) can be achieved through K rounds of cellular staining, in combination with N decoding cycles by M color types.

### Characterization of high-density and high-sensitivity MIST array in detection

The MIST array is comprised of a monolayer of 2 μm microbeads in an area of 1 cm by 1 cm, which was facilely fabricated by attaching the oligo DNA-coated microbeads to a pressure-sensitive adhesive tape covered on a plain glass side (Supplementary Fig. 1)^39^.This is a super-compact microarray that contains ~25 million DNA-coated microbeads of 50 different types. Each section of 75 μm × 75 μm imprinted by the PDMS microwells encloses 1012 ± 74 oligo DNA-coated microbeads in average (Fig. 1b). The microbeads with the same type of oligo DNA have 19 ± 4 copies per array (Fig. 1c), and this copy number variation of microbeads has been demonstrated to have negligible effect on the reproducibility and multiplexed detection of proteins in our previously reported work^38^.

The orthogonality of the 50 oligo DNA pairs was fully examined, which shows less than 1% cross reactivities between any of noncomplementary pairs (Supplementary Fig. 2). The sensitivity of those DNAs on the MIST array has been also quantified through the hybridization of complementary oligo-dyes (Fig. 1d). The sensitivity has been significantly improved by the current coating method where poly-L-lysine mediates the DNA absorption on the microbeads^39^. It is found that all oligo DNAs perform similarly in capturing their complementary oligos on the array. The averaged limit of detection (LOD) for all the 50 types of DNA microbeads is found to be around 4 pM. In consideration of the small-size microwells at 225 pL, theoretically ~542 complementary oligo copies released from antibodies binding to a single cell can be detectable.

The high quality of the MIST array ensures high fidelity of 4-cycle decoding process to assign the right colors to each microbead. In each cycle, a cocktail of complementary oligo-dyes is applied to the MIST array where 3-color fluorescent dyes are used in this report. The signal-to-noise ratio of complementary oligo-dyes labeled microbeads is generally above 5.5, and <2.3% microbeads have been incorrectly assigned with colors in the decoding test. Multiple cycles of hybridization and dissociation has no noticeable influence on the freely accessible DNAs on the microarray (Fig. 1e), which implies even more cycles possibly available when higher multiplexity is demanded. Figure 1f shows typical resultant images of the protein detection and decoding on microbeads for the detection region. Protein signals are confined within a 75 μm × 75 μm area, and the varied brightness indicates different protein expression levels. In the same region, successive decoding results of 4 cycles are shown in 3 colors for Alexa Fluor 488 (green), Cy3 (yellow) and Cy5 (red) channels, where each microbead exhibits an ordered color change over the cycles. The microbeads with the same identities are grouped, and their fluorescence intensities are quantified and averaged to generate the protein expression profile for a cell.

### Robustness of UV cleavage and reiterative staining of complementary oligo-antibody conjugates

The cross-reactivity between antibodies in each detection round was fully validated. Images in Supplementary Fig. 3 shows immunostaining of the proteins where the subcellular locations are consistent with expectation and are not affected by the presence of other antibodies in the same panel. The quality of the complementary oligo-antibody conjugates is critical to the CycMIST assays. Testing on a few crosslinking methods results in the selection of Azido/DBCO click chemistry due to high conjugation yield and convenience (Supplementary Fig. 4). To increase cleavage efficiency, two UV-cleavable nitrobenzyl groups are used instead of one in the similar conjugations previously published^32,35^. Each synthesized conjugate was purified by fast protein liquid chromatography (FPLC) and validated by a Nanodrop spectrum. Thorough study found the selection of the purification process significantly affects immunofluorescence staining result. Centrifugation by 100 KDa filter, which is a typical method adopted by the field, still left a non-trivial number of free DNAs in the solution as our FPLC result showed. Even the conjugates within the peak in Supplementary Fig. 4 performed quite differently. Thus, we only select the top 2/3 of the conjugate peak for all following experiments. The degree of labeling (DOL) was measured to be 2.2 to 4.1 complementary oligos per antibody across different conjugates. This DOL is normally deemed as the optimal one for antibody labeling.

**Fig. 2:**
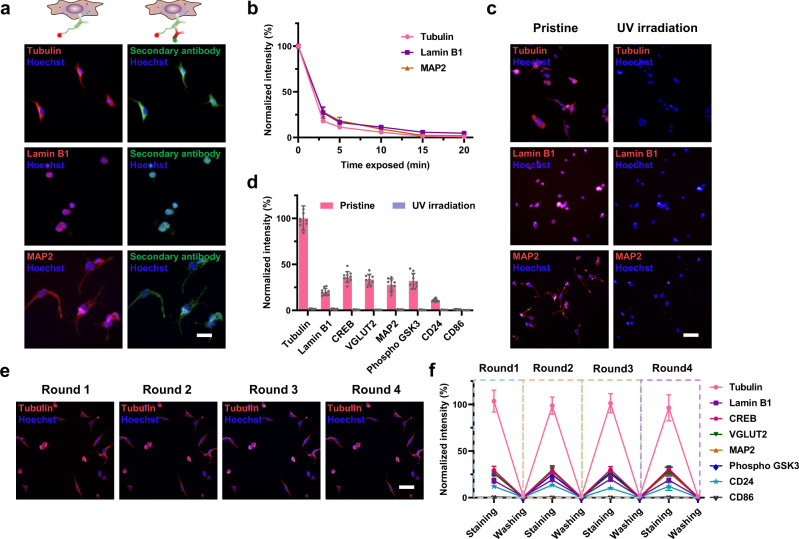
Performance of multi-round conjugates labeling and complementary oligo DNAs release. **a** Colocalization of complementary oligo-antibody conjugates immunofluorescence staining labeled with Cy5 tagged-complementary oligos (left) and labeled with Alexa Fluor-488 tagged secondary antibody (right). Scale bars: 20 μm. **b** Efficiency of UV cleavage over time for three complementary oligo-antibody conjugates, *n* = 8 independent experiments on same single cells. **c**, **d** Measurement of binding location and UV cleavage efficiency for eight conjugates, *n* = 10 independent single cells. Normalized intensity in (**d**) show ~97.4% complementary oligo being released after 15 min UV exposure. All fluorescence intensities of conjugates staining were normalized by tubulin intensity. Scale bars: 50 μm. **e, f** Reproducibility of 4 consecutive rounds of conjugates staining, releasing, and re-staining with the same complementary oligo-antibody conjugates, *n* = 10 independent experiments on same single cells. All fluorescence intensities of conjugate staining were normalized by tubulin intensity. Scale bars: 50 μm. Data are presented as mean values ± SD of more than three independent experiments, and error bars are within symbol size if not shown.

Eight conjugates for targeting Tubulin, Lamin B1, CREB, VGLUT2, MAP2, Phospho-GSK3, CD24 and CD86 that covers housekeeping protein, nucleus proteins, neuronal differentiation markers, phosphorylated protein, and cell membrane proteins have been validated by immunofluorescence staining. Some in the panel have known high expression levels, and some should have no expression. In this test, the end of complementary oligo was tagged with a Cy5 dye instead of biotin for each conjugate. The staining result is compared with that when secondary antibodies tagged with Alexa Fluor 488 dye were used. Figure 2a and Supplementary Fig. 5 show that the expected compartments of the cells are stained for all the conjugates, and the staining results by both methods are consistent. Next, we optimized UV cleavage conditions and assessed the UV-cleavage performance. With our portable UV light, cells lose fluorescence completely within 15 min exposure (Fig. 2b, c). This relatively long-time exposure should be related with UV light penetration through the thick plastic plate used for clamping and also through a layer of PDMS. Nevertheless, the cleavage has been highly efficient that no visible fluorescence is observed after UV irradiation (Fig. 2c and Supplementary Fig. 6). The quantified intensities for single cells in Fig. 2d further exhibit >97.4% of fluorescence decrease upon UV cleavage while the pre-cleavage intensities meet the expectation for those proteins.

The reiterative staining procedure has been optimized so that the same cells can be stained for multiple rounds. With this process, the antibodies binding of a later round will not be affected by the previous round, which resolved the parking issue when a large panel of antibodies is used. After each staining and imaging, the complementary oligo-antibody conjugates were dissociated by a regeneration buffer before re-staining by another cocktail of conjugates. Our result shows that the conjugates are completely removed from the cells, which is confirmed by labeling of secondary antibodies (Fig. 2e–f and Supplementary Fig. 7). Re-staining of the same cells exhibits the similar fluorescence intensities for each of the 8 conjugates with <6.5% variation. This high fidelity suggests no reduced immunogenicity of cells in the multi-round procedure, which lays the foundation for the high multiplexity of CycMIST.

### Multiplexed single-cell CycMIST for measurement of 182 proteins

**Fig. 3:**
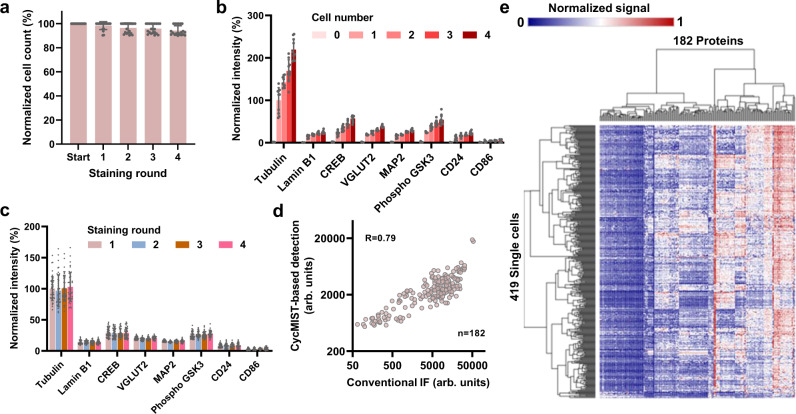
Single-cell CycMIST assay on N2a cell line. **a** Quantification of cell loss through 4 rounds of the assay on the same cells in the PDMS microwells. Error bars represent 20 times of repeats. **b** Assay sensitivity for increased number of cells loading in microwells. Error bars are standard deviations of signals from 10 independent microwells. **c** Reproducibility of CycMIST analysis of 8 proteins for 4 staining rounds on the same single cells. Error bars are standard deviations of signals from 43 single cells. **d** Correlation of 182 protein expression levels between the single-cell averages measured by CycMIST and the population levels measured by conventional immunofluorescence staining methods. The CycMIST-based signal is obtained by averaging the signal of 100 single cells. The correlation coefficient R equals 0.79 for the two methods. **e** Heatmap of unsupervised clustering result of a single-cell CycMIST assay using a whole panel of 182 proteins with 4 staining rounds. Both rows and columns are clustered by Euclidean distance and complete linkage. Data are presented as mean values ± SD of more than three independent experiments, and error bars are within symbol size if not shown. The term (arb. units) is abbreviated for arbitrary units.

The performance of the CycMIST for single-cell analysis was investigated using mouse neuroblastoma Neuro-2a cells. Cells were attached and fixed to a Pluronic® F127-coated PDMS microwells chip. Approximately only 0–2% cells were lost in each round of the experiment (Fig. 3a), which were recorded by imaging. The area of lost cells on the MIST array was excluded in data processing. The experimental protocol has been optimized so that the signals detected for each microwell area on the MIST array are pertinent to a unique cell (Supplementary Fig. 8). The 8-panel proteins are used to validate the single-cell assays. Figure 3b displays the linear increase of fluorescence signals with incremental cell numbers (0, 1, 2, 3 and 4 cells). This proves true signals being detected from the single cells. To validate the reproducibility of the technology, the same 8-panel proteins were analyzed for 4 rounds on the same cells in microwells. The detected signals are highly consistent with the bulk assay where tubulin is still the highest and CD86 is the lowest (Fig. 3c). The variation of detecting the same protein across different rounds is only 8.6%±1.5% by the single-cell CycMIST technology. Thus, every round of the detection can be deemed as a separate multiplexed assay, and the multiplexity can be efficiently increased by more round numbers.

The validated platform has been applied to measure 182 proteins in single neurons. This large panel includes proteins for typical phenotype markers of major brain cell types, AD hallmark pathways, neuron functions, enzymes, canonical signaling pathways (e.g., insulin signaling, calcium signaling, JAK/STAT signaling, autophagy signaling and apoptosis), and transcriptional factors. They were selected from Mouse Brain of Allen Brain Atlas, AlzPathway and publications related with 5xFAD mouse model (Supplementary Data 2)^40–42^. All the antibodies targeting the relevant proteins were conjugated with their corresponding complementary oligos, and the whole set was divided into 4 panels for 4 staining rounds of CycMIST experiments (Supplementary Data 1). The antibodies that can potentially interfere with each other (i.e., affinity to the protein in the phosphorylated vs. unphosphorylated state) were intentionally separated in different panels to alleviate the antibody parking issue (Supplementary Fig. 9). The averaged single-cell result for each protein was compared with that by conventional bulk assay through immunofluorescence staining (Fig. 3d). The results were relatively comparable to each other, despite that they are based on two different assays. The discrepancy could be caused by the different kinetics of the oligos released from conjugates binding to the MIST array and by the variable influence of conjugation on the antibody affinity. Both cells and proteins from the single-cell assay were clustered into groups (Fig. 3e). It was found that the surface markers are clustered on the left of the heatmap, while enzymes and essential cellular proteins are clustered on the right with higher expression. This is expected because the N2a cell line does not express a broad spectrum of brain cell markers in a dish, whereas enzymes and cytoskeleton relevant proteins are always abundant in typical animal cells. Autophagy-related 5 (ATG5), HMG synthase 1 (HMGCS1) and HMG-CoA reductase (HMGCR) were apparently the highest detectable proteins for the N2a cells in this study.

### Single-cell functional proteomics study of mouse brain cells

**Fig. 4:**
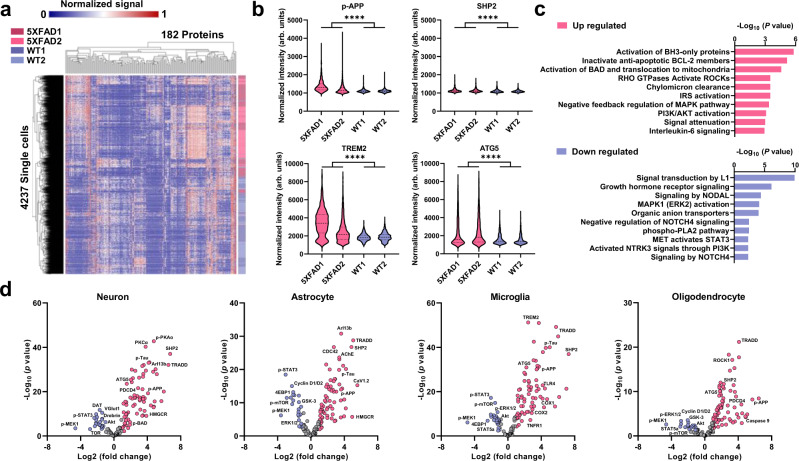
Comparison of 5XFAD and WT cortical cells through the single-cell CycMIST assay. **a** Heatmap of unsupervised clustering for both 5XFAD and WT cells. Color bar on the right indicates the distribution of the single 5XFAD cells (red) and single WT cells (purple). The single-cell data above background + 3 × SD are used for clustering by Euclidean distance and complete linkage methods. **b** Violin plots of 4 representative proteins related with AD development and comparison between 5xFAD and WT cells. ****: *p* value < 0.0001, determined by two-tailed unpaired T test. The term (arb. units) is abbreviated for arbitrary units. **c** Gene ontology analysis of upregulated proteins and downregulated proteins in 5XFAD cells when they are compared with WT cells. Pathway enrichment is expressed as − log_10_(*p* values) adjusted for multiple comparisons. **d** Volcano plots of differentially expressed proteins in 5XFAD and WT cells of the subpopulations. Red dots represent proteins expressed at high levels in 5XFAD cells, and purple dots are proteins expressed at high levels in WT cells. Y axis is − log_10_(*p* values) while X axis shows log_2_ fold change values (two-sided *T*-test unadjusted *p*  ≤  0.05 and ≥2fold change).

The single-cell CycMIST assay has been used to analyze primary mouse brain cells from two wild-type mice (WT) and two 5XFAD transgenic mice. The 5XFAD mouse model is a transgenic mouse that overexpresses the human amyloid precursor protein (APP) and human presenilin-1 containing a total of 5 familial AD mutations, and amyloid accumulation is observable at 2 months of age^43^. Neuron loss and memory deficits already occur at 6 months of age^43^. In this report, the pre-frontal cortices of the 5.5-month-old mice were surgically dissected and dissociated into single-cell suspensions containing all brain cell types, and they were immediately loaded and fixed within the PDMS microwell. The same 182 protein panel was used to analyze the cells to collect 4237 single-cell data in total (1158 cells from 5XFAD1 group, 1032 cells from 5XFAD2 group, 1046 cells from WT1 group and 1001 cells from WT2 group; Fig. 4a and Supplementary Fig. 10).

*p*-APP levels are significantly higher in 5XFAD brain cells than WT cells (Fig. 4b), which is consistent with the nature of the 5XFAD mouse model. The neurodegeneration and neuroinflammation related proteins including Src homology 2 (SH2) domain–containing phosphatase 2 (SHP2), Triggering receptor expressed on myeloid cells 2 (TREM2), and ATG5 are also expressed at a higher level in 5XFAD cells, compared to WT cells^44–46^. To visualize the protein expression profiles of single cells and compare the two samples, background noise was excluded and only the real signal of detection was subject to clustering analysis (Fig. 4a). The single-cell profiles of 5XFAD and WT cells tend to be enriched in certain sub-clusters of proteins respectively, while in some sub-clusters they are indistinguishable. To identify the individual proteins that are differentially expressed by the cells, we took advantage of large sample sizes to obtain the fold change from WT to 5XFAD for every protein with a *p* value (Supplementary Fig. 11). In general, the 5XFAD cells have more detectable proteins than the WT cells. This might be attributed to an upregulation in cellular responses to neuronal death as well as subsequent glial activation.

The single cells were further separated into neurons, astrocytes, oligodendrocytes, and microglia, according to the expression of known phenotypic markers^47–50^. The differential expression of pathway proteins is shown in Fig. 4d, where the significantly upregulated and downregulated proteins in 5XFAD cortical cells are marked in red and purple, respectively. TNFR1-associated death domain protein (TRADD), a protein reported to be associated with induction of apoptosis appears as the significantly upregulated protein in 4 cell types (neurons, astrocytes, microglia and oligodendrocytes). In microglia specifically, proteins involved in the typical neuroinflammation pathway (TLR4, COX1 and COX2) were upregulated, indicating that microglia from the 5.5-month-old 5XFAD mouse tissue display an activated phenotype, which is expected since microglia in this mouse model are known to be activated by as early as 10 weeks^51^. Cell growth and division pathways in the 5XFAD model were found to be generally downregulated, and the apoptosis pathway is upregulated (Fig. 4c), which indirectly validates our assay method.

## Discussion

The CycMIST assay enables the estimation of the protein amount in single cells, which is a distinctive feature not achievable by conventional immunofluorescence staining. Since a conjugate is labeled with ~3 complementary oligos in average and each microwell is 225 pL, the estimated LOD for protein detection is 180 protein copies per cell, which is comparable to other multiplexed single-cell technologies^24,27,31,35^. The high sensitivity is attributed to the high-density DNA on the MIST array and the small size of the microwells. However, this estimation must assume most of proteins are accessible for antibody binding, and the protein and the antibody are 1:1 ratio. For that reason, we have not converted the single-cell detection signals to protein quantities yet.

The 182 proteins selected in the assay are highly diversified: they are distributed in various compartments of a cell with varied functions. The single-cell assay results show high heterogeneity in the expression of those proteins for both N2a cell line and mouse tissue-derived cells. Just like other typical single-cell omics technologies, the tissue needs to be digested to isolate cells before the analysis. Although an optimized protocol was used to obtain single cells from mouse brains, most likely a good portion of the axon and dendrites were lost and only soma was analyzed. This is still better than typical single-cell transcriptomics techniques that isolate the nuclei for sequencing. Further improvement of our technique will rely on a further modification of the tissue dissociation protocol and even no dissociation to retain the spatial information. Alternative enzymes for digestion and other neuron dissociation kits should be considered to optimize the procedure. Nevertheless, we were still able to capture certain upregulated proteins in cells dissociated from 5XFAD mouse tissue, which is in line with what is known in the literature about pathways upregulated in the AD brain. Our assay shows that the typical proteins elevated in AD pathology are expressed at statistically higher levels in the 5XFAD mouse cells, like p-APP and certain glial activation markers, while proteins related to cell growth and division signaling are suppressed. Through gene ontology analysis (Fig. 4c), apoptotic signaling (e.g., BH3-only protein activation and BAD translocation to mitochondria) is highly up-regulated in the 5XFAD groups. Massive neuronal death due to apoptosis is a common characteristic in the brains of patients suffering from Alzheimer’s disease^52^. Besides, it was found the ROCKs signaling and chylomicron clearance are significantly elevated in the 5XFAD cells. This is similar to what is reported in literature, since ROCK protein levels in neurons are known to increase together with the activated amyloid β in mild cognitive impairment of AD brains^53^. Additionally, it is known that chylomicron contains apolipoprotein B (apoB) that may be involved in encapsulation of amyloid β and lipid metabolism in early onset of AD^54,55^. Our analysis also shows that the MAPK pathway receives high negative feedback signaling that is related with PI3K regulation. This may lead to the MAPK pathway being constantly activated in our 5XFAD cells. This is consistent with the reports that MAPK pathway is frequently altered in AD development and play a key role in neuroinflammation^56^. Our assay may provide further insights of the mechanisms in MAPK participation and potential targets for treatment. Furthermore, the CycMIST assay provides the insight of pathway alternations for the subpopulations where ~20 signaling pathways out of 34 canonical pathways were chosen in our study. Although this prototype technology has not yet achieved the genome-wide study like single-cell sequencing so the discovery of new molecules is limited, it still by far covers the highest functional protein types and could complement the sequencing technologies.

In summary, we demonstrate the single-cell CycMIST technology as a functional proteome profiling tool. It breaks the bottleneck of limited multiplexity in single-cell protein measurement where only dozens of proteins are detected. Single-cell CycMIST can assay up to 50 proteins every 2.5 h and ~200 proteins in 1 day, which is about 10X faster than other similar types of technologies^25,26^. Higher multiplexity should be achievable simply by increasing the round number, since even 20 rounds of immunofluorescence staining of cells can still produce uncompromised staining result^16,18^, and for CycMIST, each round is a separate assay. Further development of the CycMIST technology will be focused on automation, data acquisition and retaining spatial information. For instance, the released oligos could be captured locally by the MIST microbeads and thus the tissue would not need dissociation before analysis. Our microchip is amenable to integration with an instrument that can handle liquids and scan the entire array. Development of advanced data acquisition will be needed to standardize the process to be like flow cytometry, which has the hardware gating system and associated software to improve data quality and lower detection noise. A higher throughput and higher multiplexity will be achievable after further development to make the CycMIST as a tool complementary to single-cell sequencing. The single-cell CycMIST technology will potentially become a routine functional proteome tool broadly applied in various biomedical fields for mechanistic studies and diagnostics.

## Methods

### Ethical statement

All the following procedures involving animals were conducted in accordance with the US National Institutes of Health Guide for the Care and Use of Laboratory Animals and were approved by the Institutional Animal Care and Use Committee (IACUC) at Stony Brook University.

### Fabrication of PDMS microwell chip

Fabrication of polydimethylsiloxane (PDMS) chips followed the conventional methods in soft lithography^57^. Briefly, a chrome photomask (Front Range Photomask) was used to pattern a layer of features with 40 µm thickness on a 4” silicon wafer (University Wafer) by photolithography and photoresist SU-8 2025 (Kayaku Advanced Materials). The resultant mold was then pretreated with trimethylchlorosilane (TMCS; Sigma Aldrich) for 30 min to facilitate PDMS separation. Afterward, a mixture of PDMS prepolymer and curing agent (Salgard 184; Dow Corning) with a ratio of 10:1 was cast on the mold. Air bubbles were removed via vacuum desiccator for 1 h, and the PDMS mixture was baked in an oven at 80 °C for 2 h. The cured PDMS elastomer was peeled off and cut into the appropriate size for further use. Each array on the PDMS chip contains thousands of microwells for cell loading, and each microwell features with a dimension of 75 μm (length) × 75 μm (width) × 40 μm (depth).

### Oligo DNA modification on microbeads

Polystyrene microbeads (2 μm; Life Technologies) with amine group were coated with poly-L-lysine (PLL; Ted Pella) first to amplify functional groups on the beads surface. Specifically, 100 μL of 2% amine-bearing microbeads was treated with 100 μL of 10 mM bis(sulfosuccinimidyl)suberate (BS3; Pierce) crosslinker solution for 20 min. Subsequently, the microbeads were washed with Milli-Q water and then mixed with 1 mL of PLL in phosphate buffered saline (PBS; pH 8.5) buffer overnight on a vortex shaker. The PLL-funtionalizaed microbeads were rinsed with PBS buffer and then reacted with a mixture of amine-ended oligo DNA (30 μL; 300 μM; Integrated DNA Technologies), BS3 (130 μL; 2 mM), DMSO (69 μL) and PBS (31 μL) buffer solutions for 4 h. Finally, the microbeads were throughly washed with Milli-Q water and resupended to the orignal concentration for further use.

### Patterning monolayer of the MIST array and characterization

All the 50 oligo DNA-functionalized microbeads were mixed in equal portion first (14 μL for each) and then mixed with 300 μL of the blank microbeads to reduce the signal overcrowding during imaging. After sonication for 10 min, 10 μL of mixed microbeads solution was pipetted onto a 0.5 cm × 0.5 cm surface area of PDMS slab and left to dry. The dried microbeads were carefully transferred onto a cleanroom adhesive tape (VWR) which was attached to a plain glass side. Afterwards, the array was sonicated for 5 min to remove the excess layers of microbeads, leading to the formation of the uniform monolayered MIST array. The MIST arrays were prepared in batches of 40 to 50, which were then stored dry at 4 °C for later use.

The MIST array was characterized by using a cocktail of Cy5-labelled complementary oligos solutions (200 nM in 3% BSA/PBS), which was applied to the array and then incubated for 1 h, followed by PBS washing and imaging under a fluorescence microscope. The intensities of each type of microbeads were measured by ImageJ (FIJI Version 1.53). The total number of the oligo -microbeads and the number of each type of oligo-microbeads for each 75 μm × 75 μm array were quantified by a MATLAB program developed in the lab. To make calibration curve for each type of oligo-microbeads, various concentrations of Cy5 tagged complementary oligo (1 pM, 10 pM, 100 pM, 1 nM and 10 nM) were added onto the MIST array, and their fluorescence intensities were quantified. A logistic fitting function was applied to each calibration curve after taking log scale of the complementary oligo concentrations. The limit of detection (LOD) for the 50 types of oligo-microbeads are calculated by extrapolating the background fluorescence intensity plus three times of its standard deviations using the fitting curve.

### Crosstalk validation of oligo DNAs

DNA sequences were designed by a Python program that sets Tm at least 50 °C, > 53% GC content, <4 internal dimers and Tm <5 °C of cross reactivity. Over 150 candidate oligo DNAs were selected and experimentally screened to narrow down to a panel of 50 oligo DNAs. The cross-reactivities of the 50 oligo DNAs were validated by using fluorophore labelled complementary oligos s. Briefly, to investigate the crosstalk between Seq *n* (*n* means the n type of DNA) with other 49 DNAs, a MIST array only containing Seq *n*-modified microbeads and blank microbeads was fabricated according to the aforementioned process. A Cy5-labelled Seq *n*’ complementary oligo solution (200 nM in 3% BSA/PBS) was added onto the array and incubated for 1 h. The array was then thoroughly washed with PBS and imaged by a fluorescence microscope, which was denoted as positive signal. On the other hand, a 200 nM cocktail solution of the other Cy5-labelled complementary oligos (expect for Seq *n*’) was applied to the array, followed by the same procedure above, and the obtained picture was denoted as crosstalk signal. The fluorescence intensities of the positive and crosstalk signals were quantitatively analyzed by ImageJ.

### Crosstalk validation of antibodies

The cross-reactivity between antibodies in each detection round was fully validated (Supplementary Fig. 3). For a particular protein X, differentiated N2a cells were stained by a complementary oligo *n’*-antibody conjugate against protein X. Then a corresponding secondary antibody labeled with Alexa Fluor 647 was applied to the cells. The fluorescence image of protein X was taken, and the cells location of the acquired image was recorded. Subsequently, the conjugate and secondary antibody were washed out from the cells by an antibody elution buffer (composed of 0.5 M L-Glycine, 3 M Urea, 3 M Guanidinum chloride in Milli-Q water, pH 2.8). After the cell incubation with a blocking buffer (10% goat serum, 2% BSA, 1 mg/mL Salmon Sperm DNA and 0.1 % Tween 20 in PBS), the conjugate against protein X and the rest of 40+ conjugates of the same round were mixed and added to the cells. Then, 200 nM oligo *n* tagged with Cy5 in the blocking buffer was applied to the cells, and the fluorescence image for the same cells under Cy5 channel was taken. We compared the Alexa Fluor 647 image, which was the ground-truth images of protein X, to the Cy5 image, which was the protein X image acquired from the conjugate mixture staining. As shown in the Supplementary Fig. 3, for all the tested antibodies in each detection round, the staining patterns of the two images overlay with each other, indicating the cell staining by the conjugate mixture bound to only their target epitopes, not to other proteins.

### Preparation of complementary oligo-fluorophore dye conjugates

Three fluorescent colors (Alexa Fluor 488, Cy3 and Cy5) of complementary oligo-dye conjugates were used for the decoding of 50 different oligo-microbeads. The Cy3- and Cy5-labelled complementary oligos were directly purchased from Integrated DNA Technologies, and the Alexa Fluor 488-labelled complementary oligos were synthesized in the lab. Briefly, 50 μL of 200 μM complementary oligos were incubated at pH 8.5 with 50 equivalents of Alexa Fluor 488 NHS Ester (Life Technologies) in 30% DMF for 4 h. Afterwards, excess chemical reagents were removed by Zeba spin desalting column (7 K MWCO; Thermofisher), with PBS as a wash buffer. The Alexa Fluor 488-labelled complementary oligos were diluted to 10 μM and stored at −20 °C for later use.

### Preparation and purification of UV-cleavable, biotinylated complementary oligo-antibody conjugates

All the antibodies (BSA-free) details and their corresponding complementary oligo barcodes used in this study were summarized in Supplementary Data 1 and Supplementary Data 2. Prior to complementary oligo conjugation, all antibodies were tested on N2a cells by conventional immunofluorescence microscopy and then compared with the information provided by vendors and literature to ensure the binding and localization of each antibody with respect to its target. Custom designed oligo DNAs were purchased from Integrated DNA Technologies and used as received.

The conjugation of biotinylated complementary oligo with antibody was achieved through click chemistry reaction. Reactions should be covered in foil or in a dark room due to the light sensitivity of UV-cleavable crosslinkers. In detail, antibodies were first concentrated to 1 mg/mL or higher using Amicon Ultra centrifugal filter (10 K MWCO; EMD) to improve the conjugation efficiency. Both antibodies and complementary oligos were then solvent exchanged to a PBS buffer with pH 8.5 by Zeba spin desalting column (7 K MWCO; Thermofisher) to remove sodium azide and reach the optimal pH for efficient amine labelling. Afterwards, 50 μL of 1 mg/mL antibodies were reacted with 1 μL of 10 mM UV-cleavable azido-NHS ester (30 equivalents to antibodies; Click Chemistry Tools) for 2 h, while 30 μL of 200 μM complementary oligos were incubated with 1.2 μL of 100 mM UV-cleavable DBCO-NHS ester (20 equivalents to complementary oligos; Click Chemistry Tools) in 30% DMF for 4 h. The azido-antibodies and DBCO-complementary oligos were buffered exchanged to pH 7.4 PBS using Zeba spin desalting column (7 K MWCO) to remove excess regents. The DBCO-complementary oligos were mixed with their corresponding azido-antibodies for reaction overnight at 4 °C, resulting in the formation of complementary oligo-antibody conjugates. The conjugates were purified using a FPLC workstation with Superdex® 200 gel filtration column at 0.5 min/min isocratic flow of PBS buffer. Only a portion of the conjugate peak is selected to ensure high complementary oligo loading to each antibody. Finally, the desired products were collected from FPLC elutions and then concentrated to 0.3–0.5 mg/mL and stored in 4 °C for further use. The absorbance spectrum of UV-cleavable biotinylated complementary oligo-antibody conjugates were measured by a Nanodrop spectrophotometer (Thermo Fisher) to determine the degree of labeling (DOL) by following equation^34^:

1 $${DOL}=\frac{\varepsilon 260{Ab}-R* \varepsilon 280{Ab}}{R* \varepsilon 280{DNA}-\varepsilon 260{DNA}}$$

Where ε260_Ab/DNA_ and ε280_Ab/DNA_ are the extinction coefficients of each complementary oligo and antibodies at both 260 nm and 280 nm, respectively. R is the absorbance ratio (260/280 nm) of the complementary oligo-antibody conjugates.

### Cell preparation and immunofluorescence staining

Mouse neuroblastoma Neuro-2a (N2a) cells (ATCC^®^ CCL-131™) were cultured in Dulbecco’s modified Eagle’s medium (DMEM; Life Technologies) containing 10% (v/v) fetal bovine serum (FBS), 100 µg/ml streptomycin sulfate, and 100 U/ml penicillin G sodium at 37 °C in a humidified atmosphere with 5% CO_2_. N2a cells were passaged twice a week with a cell density of ~80–90%. For the induction of neuronal differentiation, N2a cells were plated at a density of 2 × 10^4^/cm^2^ and maintained in the standard growth medium for 24 h. Next day, the N2a cells were stimulated by a reduced serum medium (DMEM supplemented with 2% FBS) in the presence of 20 μM Retinoic acid (RA; Sigma-Aldrich). The medium was refreshed every 24 h. The cells having one or more neurites of a length more than twice the diameter of the cells body were defined as differentiated.

Prior to cell staining, all the buffers and solutions were first warmed up to room temperature. Then the cells were rinsed with PBS buffer, fixed with 4% formaldehyde in PBS for 20 min, permeabilized with 0.1% Triton-X100/PBS for 7 min and washed three times with PBS buffer. For the conventional immunostaining by the unmodified antibodies, the fixed cells were incubated with 5% goat serum (Cell signaling) in PBS for 1 h, stained with 10 μg/mL of primary antibodies in 5% goat serum for 1 h, incubated with Alexa Fluor 488- conjugated secondary antibodies in 5% goat serum for 1 h and then stained with 5 μg/mL of Hoechst 33342 (Pierce) in PBS for 15 min. The cells were washed three times with PBS buffer between the steps. For staining by the complementary oligo-antibody conjugates, the fixed cells were incubated with a blocking buffer (10% goat serum, 2% BSA, 1 mg/mL Salmon Sperm DNA and 0.1 % Tween 20 in PBS) for 0.5 h to minimize non-specific antibody and DNA binding. After washing three times with PBS, the cells were stained with 10 μg/mL of complementary oligo-antibody conjugates in the blocking buffer for 1 h, followed by incubation with Alexa Fluor 488-conjugated secondary antibodies for 1 h and then stained with 5 μg/mL of Hoechst 33343 in PBS for 15 min. Finally, the cells were imaged under a fluorescence microscope.

### UV cleavage and cyclic immunostaining

Eight different UV-cleavable Cy5 labelled complementary oligo -antibody conjugates (anti-Tubulin/Lamin B1/CREB/VGLUT2/MAP2/Phospho-GSK3/CD24/CD86) were used to label cells according to the immunostaining procedure above. For kinetic measurements, the plates with cells were exposed to a UV light (365 nm; Thorlabs CS2010 UV Curing LED System) for different durations and then washed thoroughly with PBS buffer. Fluorescence images of the cells before and after UV irradiation under different exposure time were taken using a fluorescence microscope, and the fluorescence intensities of each image were quantitatively measured by ImageJ.

Cyclic immunostaining was performed in a parallel and sequential manner in 96-well plates. Briefly, for each of the eight conjugates, each staining round consisted of four steps: 1) blocking; 2) staining; 3) imaging; 4) washing. The first three steps were identical to the staining procedure as described above. The washing step was achieved by incubating the stained cells with 200 μL of regeneration buffer (composed of 0.5 M L-Glycine, 3 M Urea, 3 M Guanidinum chloride in Milli-Q water, pH 2.8) three times for 2 min each^16^. Afterwards, the cells were stained with Alexa Fluor 488-conjugated secondary antibodies to verify that the conjugates were completely eluted from the cells. Following the washing step, the cells were washed with PBS and incubated with the blocking buffer for 1 h to proceed the next staining round. This staining round was repeated four times to complete the cyclic immunostaining. Fluorescence images of the cells after each staining and washing step of the four cyclic staining rounds were recorded and quantitatively analyzed.

### Multiplexed single-cell protein detection and decoding process

The PDMS microwell were first treated with a plasma cleaner (Harrick Plasma) for 1 min and then incubated with 0.5% Pluronic® F-127 (Sigma Aldrich) in Milli-Q water for 30 min to reduce nonspecific conjugate adsorption on the surface. After cleaning with PBS, a 200 µL of cell suspension with a concentration of 30,000 cells/mL in culture medium was applied onto the microwell chip for 7 min to load the cells, and the PDMS slab was then placed on a shaking table with a speed of 40 rpm/min. The excess cells that were not attached to the microwell surface were gently removed by pipetting. The cell loading result follows Poisson statistics, in which almost 19.8%±3.2% of microwells could be occupied by individual cells (Supplementary Fig. 12). The cells inside of the microwells were stained with a cocktail of UV-cleavable biotinylated complementary oligo-antibody conjugates according to the immunostaining protocol as described above. After the completion of the staining, the chip was washed three times with PBST containing 0.1% Tween 20 and 5% goat serum. A glass slide carrying a MIST array was carefully mated with the PDMS chip on a clamp to completely isolate the cells in the microwells. The whole device was exposed to a UV light for 15 min to release the biotinylated complementary oligos and then left for another 1 h to allow the hybridization of complementary oligos with oligo on the MIST array. The MIST array was then separated from the PDMS microwells and washed with 3% BSA in PBS. 150 μL of 10 μg/mL streptavidin-Alexa Fluor 647 dye (Life Technologies) in 3% BSA/PBS solution was added on the MIST array for signal visualization, which was corresponding to protein signal. Meanwhile, the cells in the PDMS microwells were washed with the regeneration buffer for the next staining round by another different cocktail of conjugates.

The fluorescence images of the protein signal on the MIST array were recorded and then proceeded to the decoding process. The decoding procedure consisted of four cycles using three different colors of complementary oligo-dyes, which can identify maximum 3^4^ (81) types of microbeads/proteins. For the decoding of the first cycle, 150 μL of 2 M NaOH solution was applied onto the array to dissociate double stranded DNAs for 1 min. After washing with saline-sodium citrate buffer (SSC) for three times, 150 μL of a cocktail of complementary oligo-dye conjugates (Cocktail I, 200 nM) in hybridization buffer (40% formamide and 10% dextran sulfate in SSC buffer) was added on the array and incubated for 1 h. The array was washed with SSC buffer and subsequently imaged under a fluorescence microscope, which was denoted as Cycle 1 signal. The procedures of the second, third and fourth decoding cycles were similar to that of the first cycle, except that Cocktail II, Cocktail III and Cocktail IV of complementary oligo-dye conjugates were used to label the array, resulting in the decoding signals of Cycle 2, Cycle 3 and Cycle 4, respectively. All the images of the Cycle 1 signal to Cycle 4 signal were registered together to determine the order of fluorescent color change of each microbead by a Matlab program developed in the lab.

### Mouse brain sample preparation

All the following procedures were conducted in accordance with the US National Institutes of Health Guide for the Care and Use of Laboratory Animals and were approved by the Institutional Animal Care and Use Committee (IACUC) at Stony Brook University. Brains of two male wild-type (WT) and two male C57BL/6 (5XFAD) transgenic mice with ages of 5.5-month-old were used in this study. The mice were housed in ventilated cages under standard conditions for mice (temperature between 20–26 °C, humidity range 40–50%, a 12 h light/12 h dark cycle) with ad libitum access to food and water. The mice from WT and 5xFAD models were cervically dislocated, and the brains were removed from the skull. For each mouse, the prefrontal cortices (PFC) were dissected out from both hemispheres since those are the regions most affected by amyloid plaque pathology. The dissected PFC were proceeded with tissue dissociation using the commercial Miltenyi Biotec Adult Brain Dissociation Kit (ADBK), which involves debris clearance and red blood cell removal steps. In detail, the PFC samples were cut into small slices and immediately transferred into gentleMACS C-tube (Miltenyi) containing ADBK enzymatic digest reagents. The samples were dissociated using the gentleMACS Octo Dissociator instrument (Miltenyi) under the “37C_ABDK” program with heaters attached. After sample digestion, cell suspension was filtered for debris clearance by 70 µm MACS SmartStrainer (Miltenyi) with ice-cold Dulbecco phosphate buffered saline (DPBS). Finally, the cell suspension was processed for red blood cell removal following manufacturer’s suggested centrifugation procedure and buffer solutions provided. An estimated number of 600,000 cells were finally obtained by cell counting, and a diluted cell suspension with a concentration of 30,000 cells/mL was used to load into PDMS microwell chips for subsequent CycMIST assay.

### Image quantification and registration

A Nikon Ti2 inverted fluorescence microscope equipped with a motorized stage was used to automatically take images for the entire PDMS microwells and the MIST array. Bright-field and phase-contrast images were utilized to identify the microwell address and microbead locations. For the fluorescence images on the Hoechst, Alexa Fluor 488, Cy3 and Cy5/Alexa Fluor 647 channels, the light were filtered with beam splitters and emission filters controlled by a wavelength switcher. All the images were saved as the format of 16-bit ND2 in the Nikon software (NIS-Elements AR Microscope Imaging Software) and then were passed to MATLAM program for further registration and processing.

An in-house MATLAB program code was utilized for image overlapping, registration and signal analysis. The digitalized images for each dataset on a 75 μm × 75 μm array composed of protein signal images, bright-field images and three different color images from the four decoding cycles (Supplementary Fig. 13). Bright images from protein signal were read first as a reference to generate registration information, and the same registration information were used to align images from the protein signal and four decoding cycles. All the aligned images were then compiled into multi-image stacks to identify the order of fluorescent color change for each microbead and quantify the fluorescence intensity of individual microbead on the protein signal. The protein ID of each microbead was determined by comparing the color order with the proteins in the decoding design (Supplementary Data 1). The microbeads detecting the same protein were grouped and their fluorescence intensities were averaged for each 75 μm × 75 μm array. Meanwhile the microwells of the entire chip were scanned to record the cell number and location of each microwell. After compiling the information of cell number counting on each microwell or microbead array, a final dataset was produced including the results of cell number (zero or one cell) and the proteins detection signal and their corresponding fluorescence intensities.

### Data analysis

For single cells analysis, only the microwells with one cell was used for signal collection and analysis, and the detected protein signal in the microwells of zero cell was taken as the background signal. The mean intensity plus 3 × standard deviation of the background signals was considered as the confident threshold, and the proteins signals of each microbead array above the threshold were regarded as the true signals of the detected proteins. Unpaired, 2-tailed T-test was used to evaluate the statistical significances with GraphPad Prism 8.0, which were considered at the *p* value < 0.0001 (****) levels. The same software was used to generate violin plots. Heatmaps and unsupervised clustering were generated with the Morpheus program (Broad Institute, Morpheus, https://software.broadinstitute.org/morpheus). The univariate analysis of protein intensities for WT and 5XFAD samples was based on fold-change values and the threshold of significance by building a volcano plot (setting: p-values ≤ 0.05, fold change ≥2.0. The four subpopulations of WT and 5XFAD were separated by the expression of their surface markers including FOX3 for neurons, GFAP for astrocytes, Oligodendrocyte Marker O1 for oligodendrocytes and CD11b for microglia^47–50^. The single cells expressing these markers were separated by k-means at 4 groups with the Morpheus program, and the intensities of the cells below the threshold (background mean intensity plus 3 × SD) of those markers were not counted. Within each subpopulation, the same univariate analysis of protein intensities for 5XFAD and WT cells was conducted to differentiate proteins upregulated and downregulated in the 5XFAD sample. Gene ontology (GO) enrichment analysis of the generated datasets of differentially expressed proteins from canonical pathways was performed via the GO web pages at http://www.geneontology.org/. The hypergeometric test after the false discovery rate (FDR) correction was used to assess statistical significance. Enriched GO terms with FDR-corrected *p* < 0.05 were considered statistically significant. Top 10 significant pathways from the upregulated proteins and downregulated proteins in 5XFAD cells were identified using the Reactome pathway analysis.

### Statistics and reproducibility

All the measurements were performed at least three duplicates (n ≥ 3) and the data were presented as mean ± SD (standard deviation). The imaging results (Fig. 2a, c, e and Supplementary Fig. 1a; 3b; 5; 6; 8a, b) were repeated more than three times, respectively, with similar results.

### Reporting summary

Further information on research design is available in the Nature Research Reporting Summary linked to this article.

## Supplementary information

Supplementary information

Reporting Summary

Description of Additional Supplementary Files

Supplementary Data 1

Supplementary Data 2

Supplementary Software

## Data Availability

All the data supporting the study are available within the article and its Supplementary Information files. Source data are provided with this paper.

## Source data

Source Data

## Code availability

The image processing code written in MATLAB is provided in Supplementary Software along with instruction.
