# Supplementary material for: Cyclic microchip assay for measurement of hundreds of functional proteins in single neurons
Source: Nat Commun. 2022 Jun 21;13:3548. doi: 10.1038/s41467-022-31336-x (PMC9213506; doi:10.1038/s41467-022-31336-x)
Supplement: Supplementary file 1 — Supplementary information [file 41467_2022_31336_MOESM1_ESM.pdf]

Supplementary information for

Cyclic Microchip Assay for Measurement of Hundreds of Functional Proteins in Single Neurons

Liwei Yang[1], Avery Ball[1], Jesse Liu[1], Tanya Jain[2, 3], Yue-Ming Li[2, 3, 4], Firoz Akhter[5], Donghui Zhu[5], Jun Wang[1#]

[1]Multiplex Biotechnology Laboratory, Department of Biomedical Engineering, Stony Brook University, Stony Brook, NY 11794

[2]Chemical Biology Program, Memorial Sloan Kettering Cancer Center, New York, NY, USA

[3] Programs of Neurosciences and [4]Programs of Pharmacology, Weill Graduate School of Medical Sciences of Cornell University, New York, NY, USA

[5] Department of Biomedical Engineering, Stony Brook University, Stony Brook, NY, USA, 11794

[#]Corresponding authors. E-mail address: Jun.wang.5@stonybrook.edu

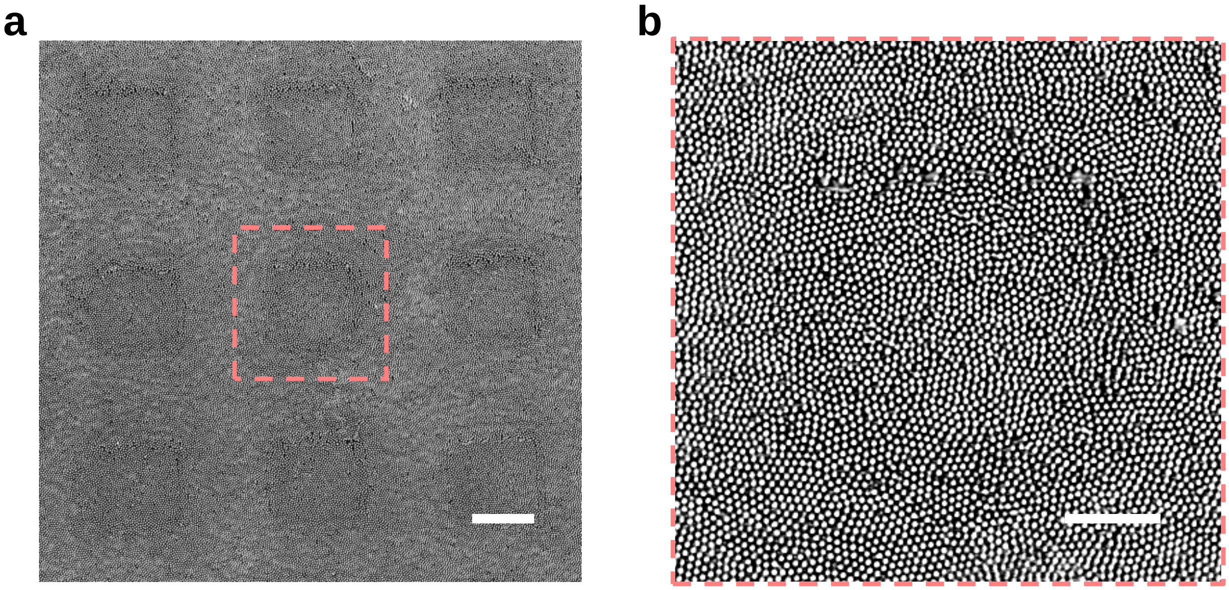

Supplementary Figure 1. a) Bright field images of a large-scale microbeads array after clamping with a PDMS microwell chip, scale bar = 50 μm. b) Zoom-in images of the red dotted frame in Supplementary Figure 1a, scale bar = 20 μm.

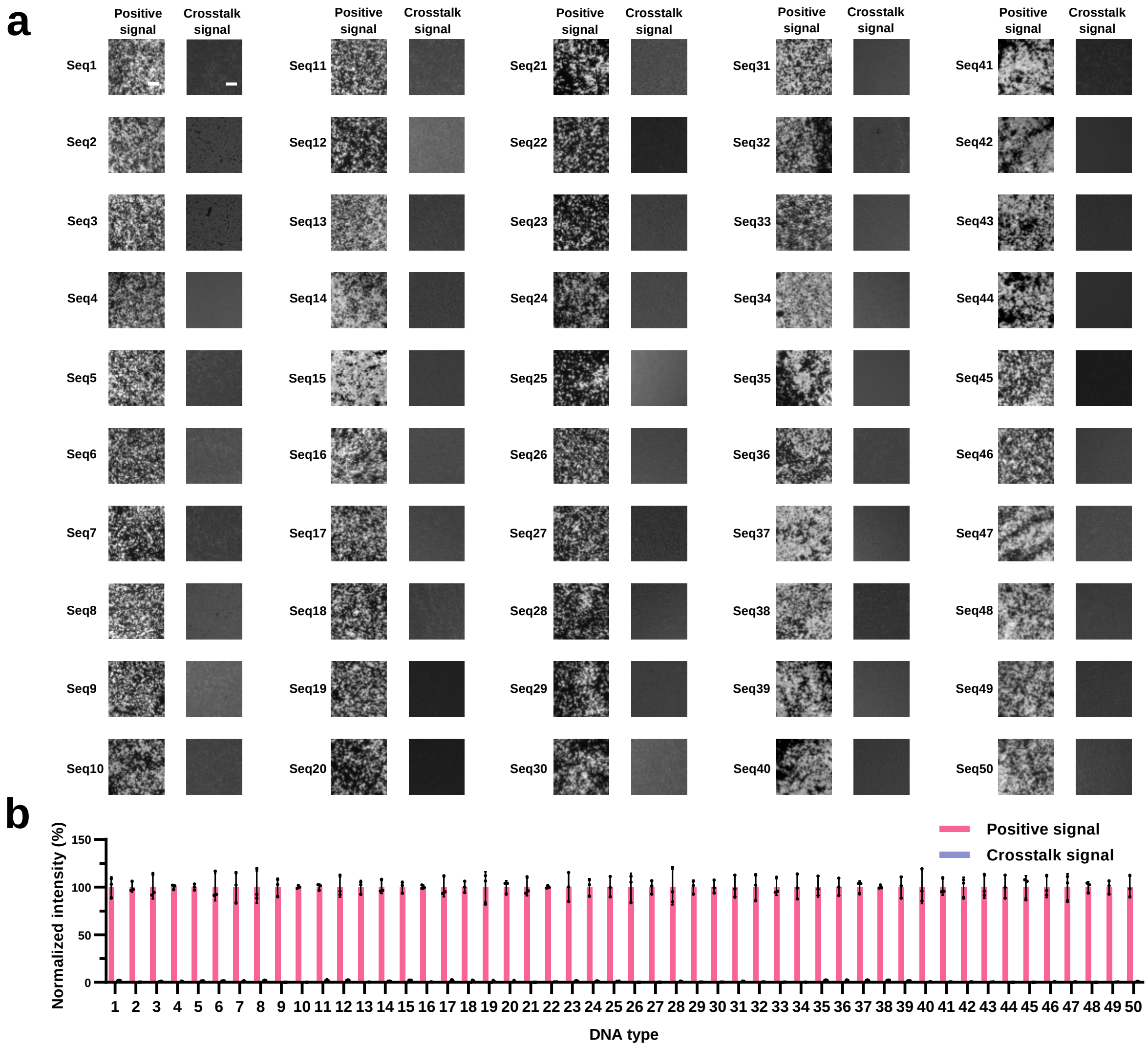

Supplementary Figure 2. Crosstalk validation of 50 different types of oligo DNAs. a) Fluorescence images of positive signal and crosstalk signal for each tested DNA. b) The fluorescence intensity of each image in Supplementary Figure 2a, which were analyzed quantitatively using the software of ImageJ and normalized by the intensity of positive signal for Seq1 DNA. Data are presented as mean ± SD, n = 3 independent areas. Error bars are within symbol size if not shown.

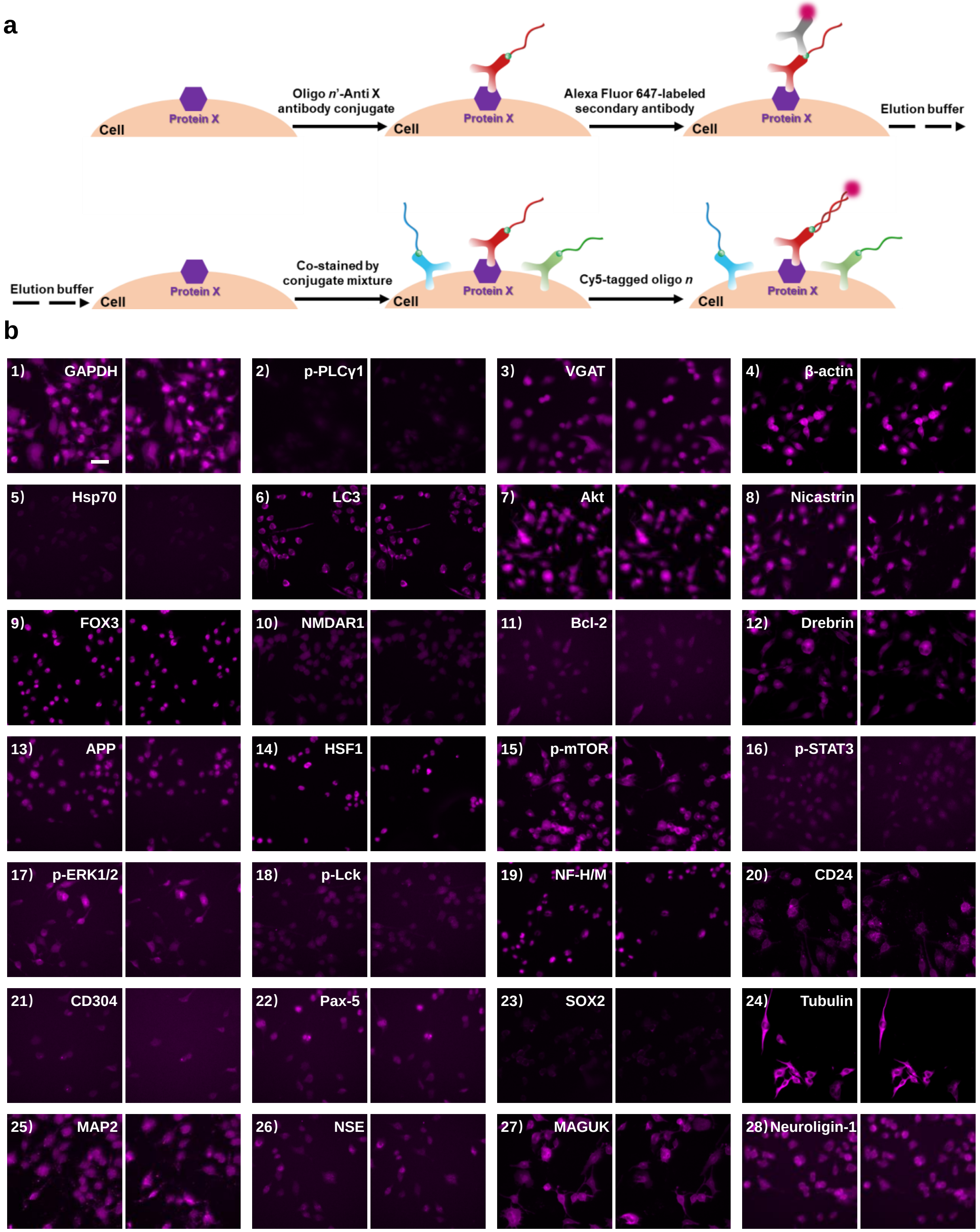
a
Cell
Protein X
Oligo n'-Anti X antibody conjugate
Alexa Fluor 647-labeled secondary antibody
Elution buffer
Co-stained by conjugate mixture
Cy5-tagged oligo n
b
1) GAPDH
2) p-PLCγ1
3) VGAT
4) β-actin
5) Hsp70
6) LC3
7) Akt
8) Nicastrin
9) FOX3
10) NMDAR1
11) Bcl-2
12) Drebrin
13) APP
14) HSF1
15) p-mTOR
16) p-STAT3
17) p-ERK1/2
18) p-Lck
19) NF-H/M
20) CD24
21) CD304
22) Pax-5
23) SOX2
24) Tubulin
25) MAP2
26) NSE
27) MAGUK
28) Neuroligin-1

Continued

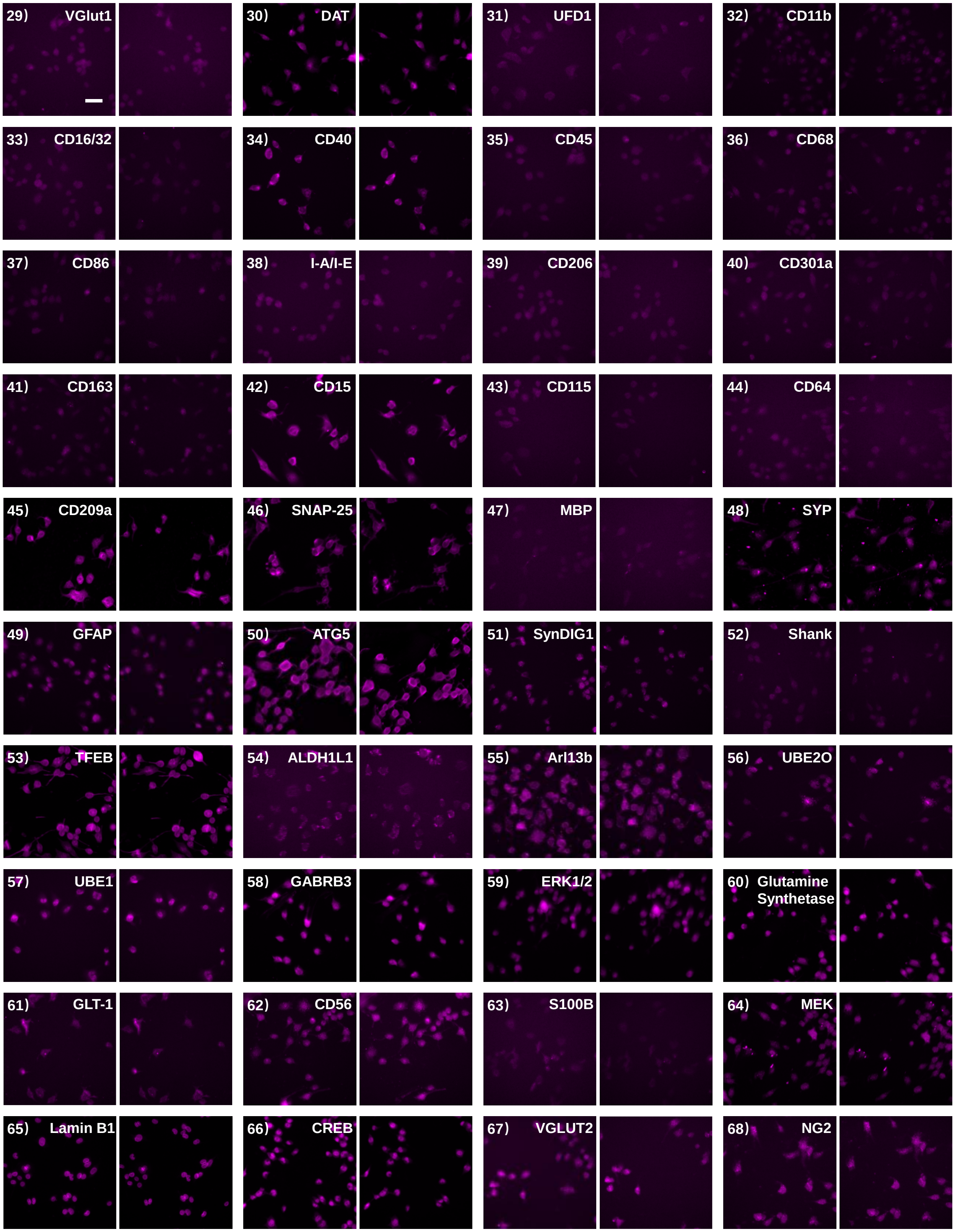

29) VGlut1
30) DAT
31) UFD1
32) CD11b
33) CD16/32
34) CD40
35) CD45
36) CD68
37) CD86
38) I-A/I-E
39) CD206
40) CD301a
41) CD163
42) CD15
43) CD115
44) CD64
45) CD209a
46) SNAP-25
47) MBP
48) SYP
49) GFAP
50) ATG5
51) SynDIG1
52) Shank
53) TFEB
54) ALDH1L1
55) Arl13b
56) UBE2O
57) UBE1
58) GABRB3
59) ERK1/2
60) Glutamine Synthetase
61) GLT-1
62) CD56
63) S100B
64) MEK
65) Lamin B1
66) CREB
67) VGLUT2
68) NG2

Continued

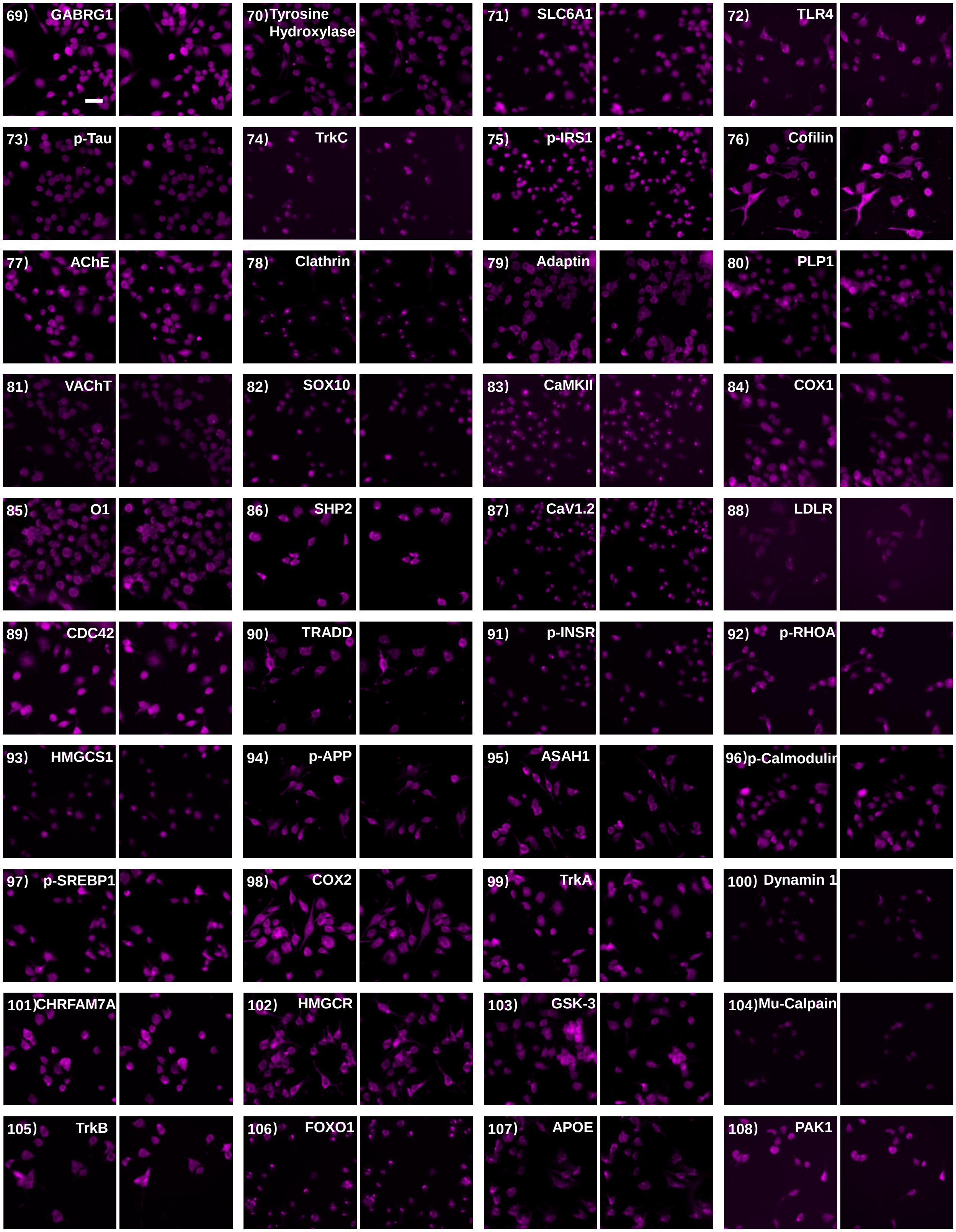
69) GABRG1
70) Tyrosine Hydroxylase
71) SLC6A1
72) TLR4
73) p-Tau
74) TrkC
75) p-IRS1
76) Cofilin
77) AChE
78) Clathrin
79) Adaptin
80) PLP1
81) VAChT
82) SOX10
83) CaMKII
84) COX1
85) O1
86) SHP2
87) CaV1.2
88) LDLR
89) CDC42
90) TRADD
91) p-INSR
92) p-RHOA
93) HMGCS1
94) p-APP
95) ASAH1
96) p-Calmodulin
97) p-SREBP1
98) COX2
99) TrkA
100) Dynamin 1
101) CHRFAM7A
102) HMGCR
103) GSK-3
104) Mu-Calpain
105) TrkB
106) FOXO1
107) APOE
108) PAK1

Continued

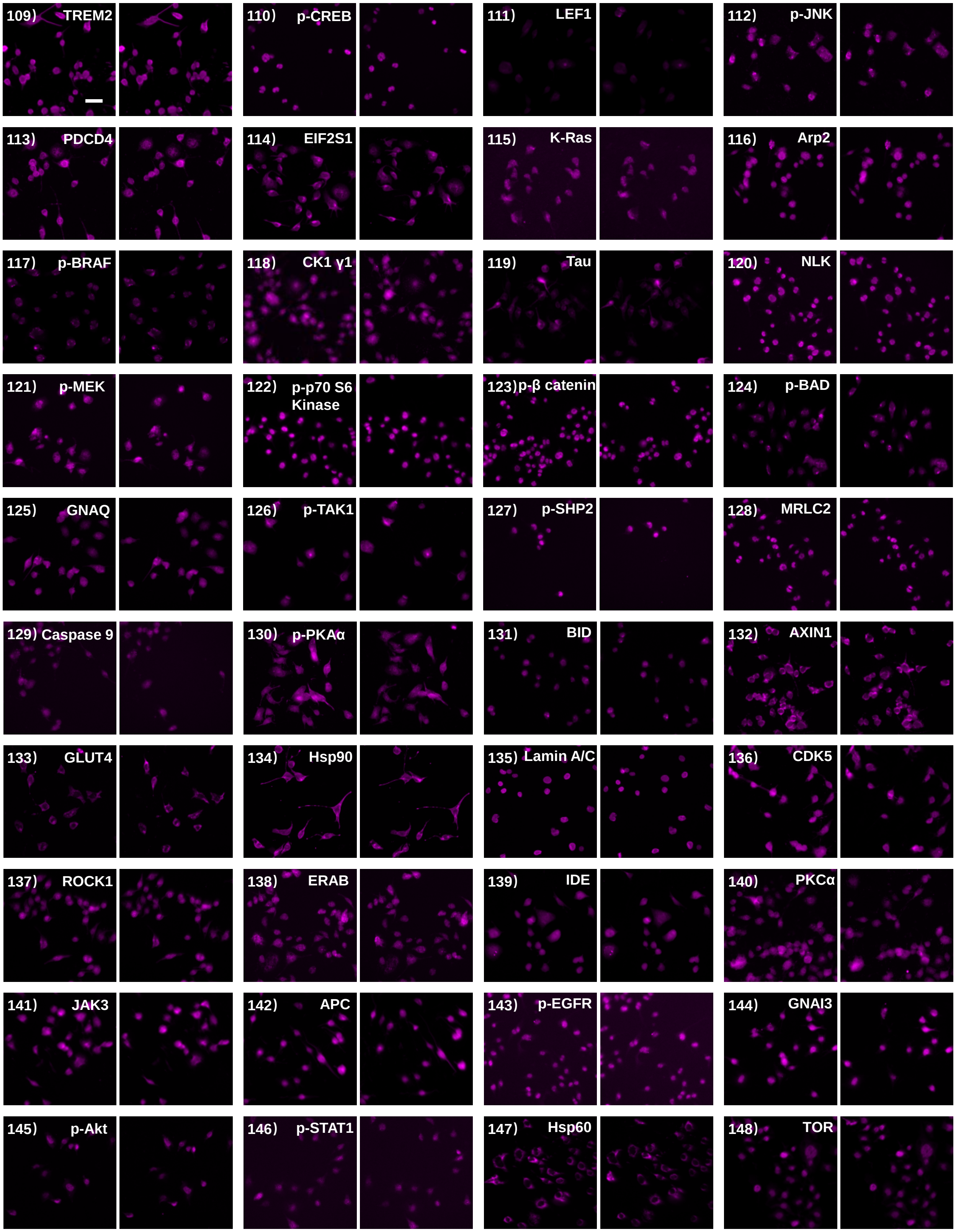
109) TREM2
110) p-CREB
111) LEF1
112) p-JNK
113) PDCD4
114) EIF2S1
115) K-Ras
116) Arp2
117) p-BRAF
118) CK1 γ1
119) Tau
120) NLK
121) p-MEK
122) p-p70 S6 Kinase
123) p-β catenin
124) p-BAD
125) GNAQ
126) p-TAK1
127) p-SHP2
128) MRLC2
129) Caspase 9
130) p-PKAα
131) BID
132) AXIN1
133) GLUT4
134) Hsp90
135) Lamin A/C
136) CDK5
137) ROCK1
138) ERAB
139) IDE
140) PKCα
141) JAK3
142) APC
143) p-EGFR
144) GNAI3
145) p-Akt
146) p-STAT1
147) Hsp60
148) TOR

Continued

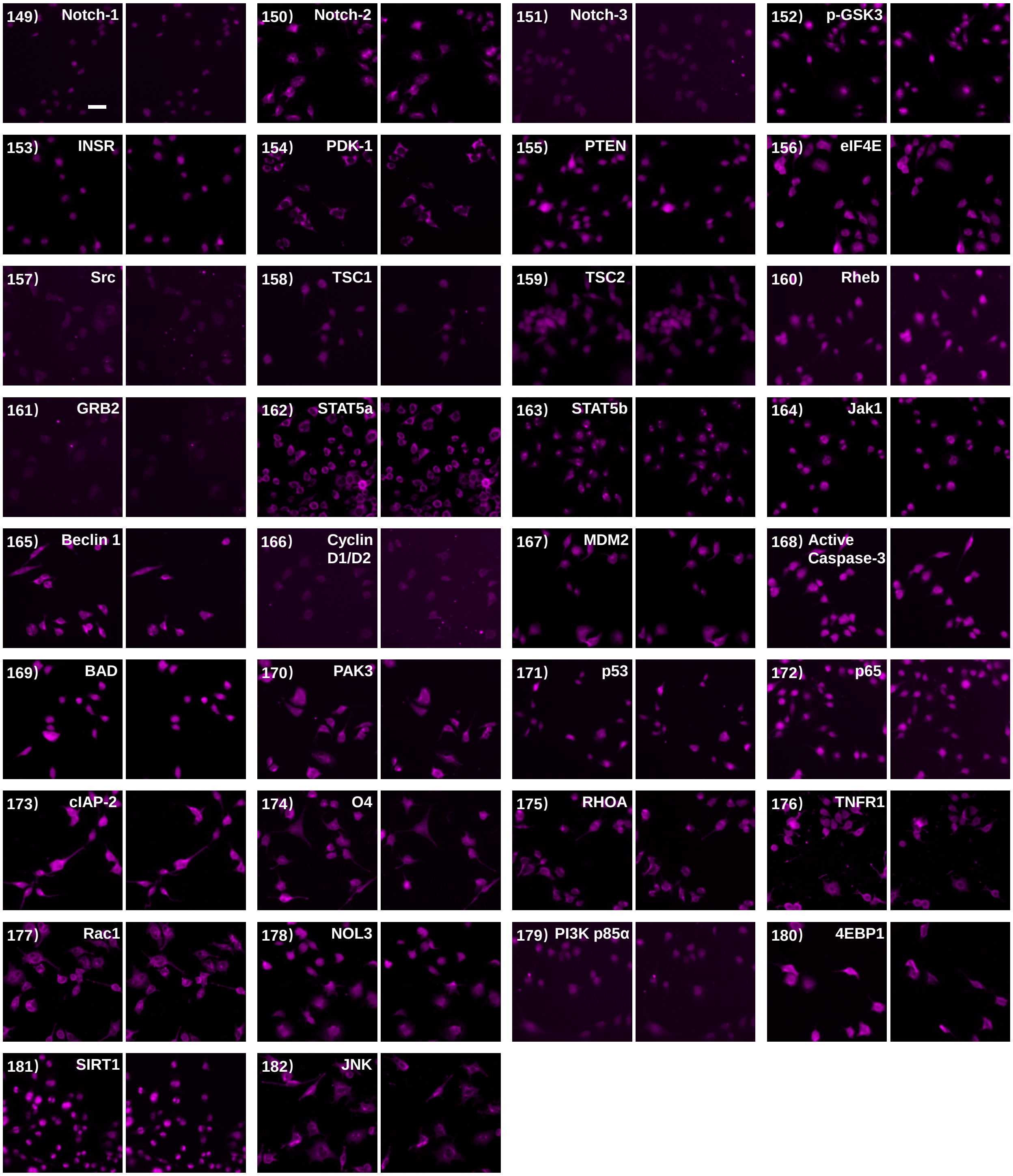

Supplementary Figure 3. a) Schematics of the antibody cross-reactivity validation experiments. b) Fluorescence images of ground-truth protein signal with single oligo-antibody conjugate being used (Alexa Fluor 647 channel, left), and fluorescence signal with labeling by a Cy5-tagged oligonucleotide while a mixture of complement oligo-antibody conjugates was applied to the same cells (Cy5 channel, right). The images of cells were taken under the same location where some cells might lose due to multiple washing steps. No crosstalk among the conjugate mixture in each detection round was observable. Scale bar = 50 µm.

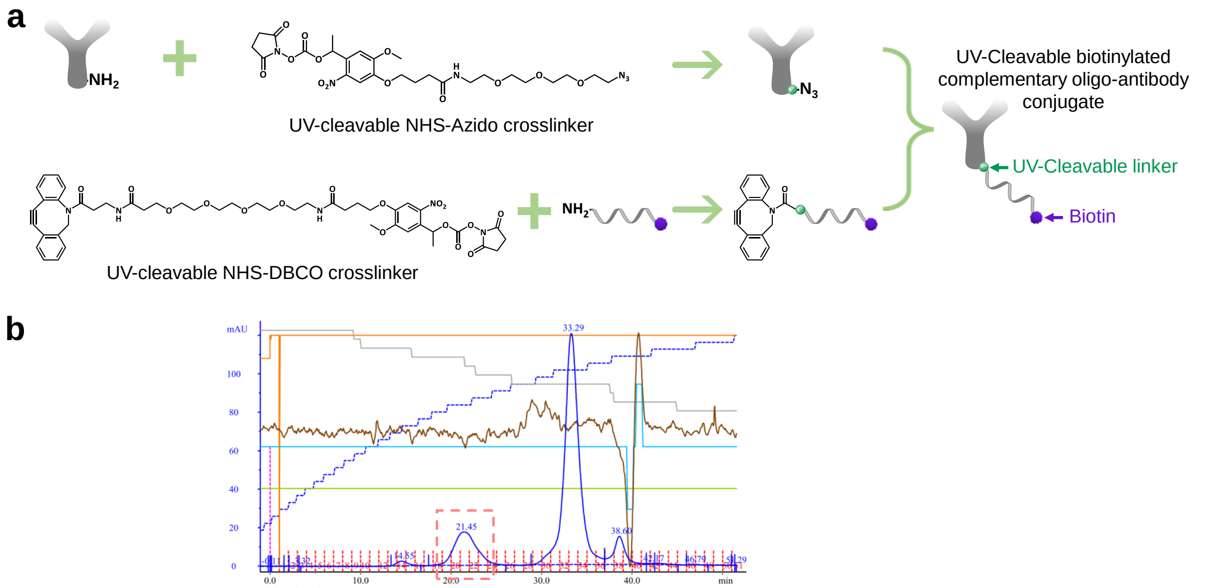

Supplementary Figure 4. a) Synthetic scheme for the preparation of UV-cleavable biotinylated complementary oligo-antibody conjugates. b) Typical FPLC purification curve of the conjugates, the red dotted frame indicates the peak of the purified conjugate, where the location in the chromatography is correlated with the number of chemical loads.

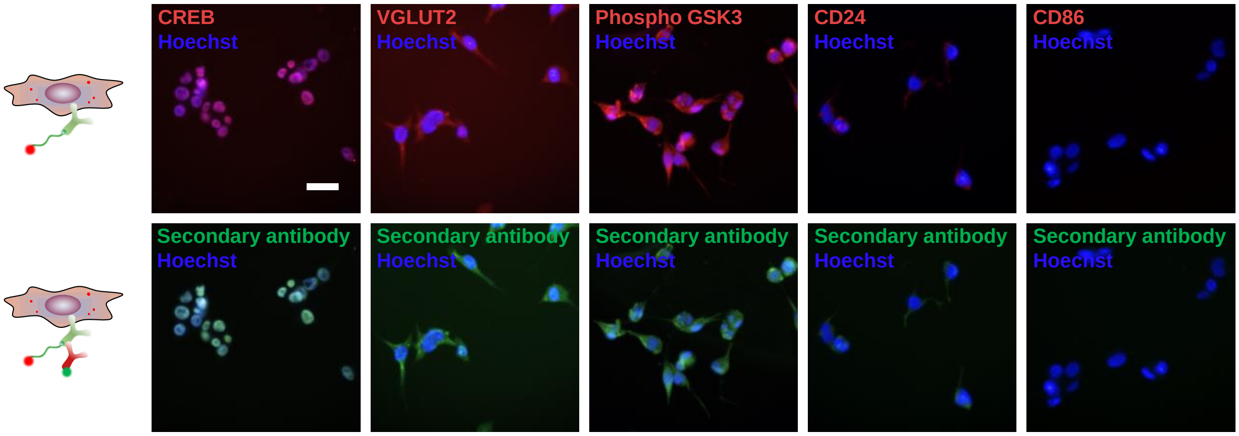

Supplementary Figure 5. Validation of conjugates staining specificity. The up-panel: fluorescence images of the differential N2a cells after incubation with Cy5-labeled complementary oligo conjugated to anti-CREB/VGLUT2/Phospho-GSK3/CD24/CD86 antibodies. The bottom-panel: fluorescence images of the stained cells after incubation with Alexa Fluor 488-labelled secondary antibodies. Scale bar = 20 μm.

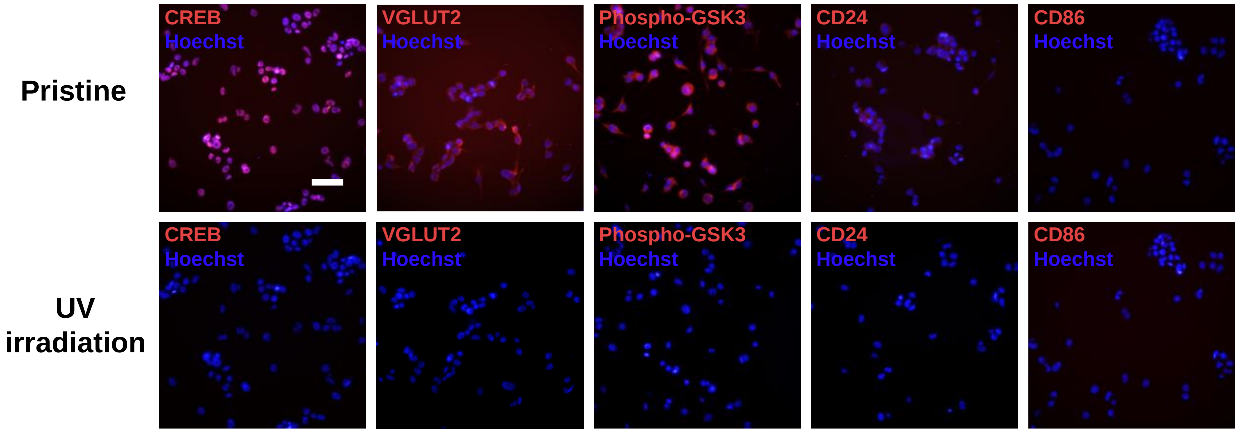

Supplementary Figure 6. UV-cleave efficiency of conjugates. The up-panel: fluorescence images of the differential N2a cells after incubation with Cy5 labeled complementary oligo conjugated to anti-CREB/VGLUT2/Phospho-GSK3/CD24/CD86 antibodies. The bottom-panel: fluorescence images of the stained cells after 15 min of UV-light irradiation, demonstrating that complementary oligo barcodes were released from the stained cells. Scale bar = 50 μm.

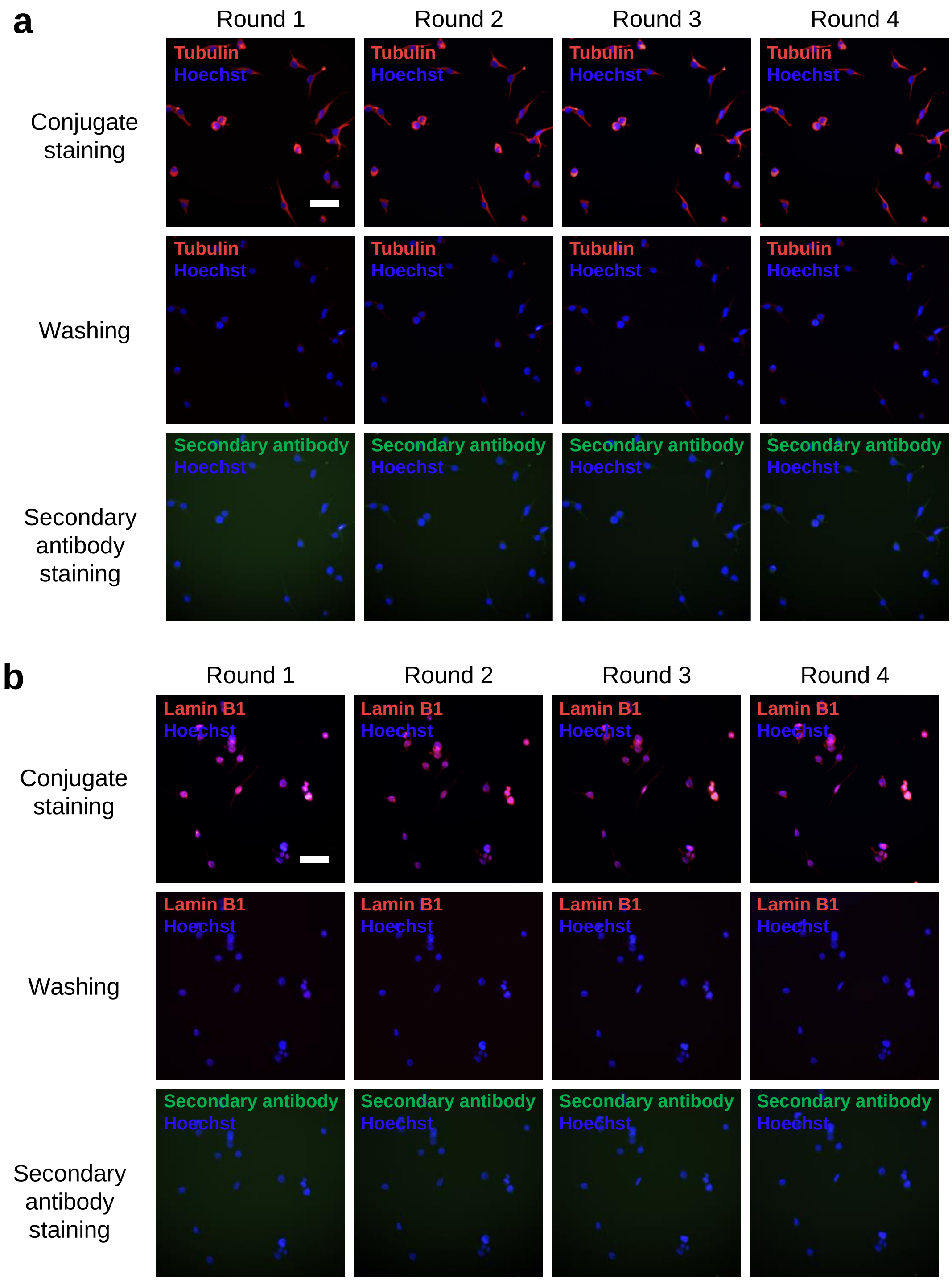
a
Round 1
Round 2
Round 3
Round 4
Conjugate staining
Washing
Secondary antibody staining
Tubulin
Hoechst
Secondary antibody
b
Round 1
Round 2
Round 3
Round 4
Conjugate staining
Washing
Secondary antibody staining
Lamin B1
Hoechst
Secondary antibody

Continued

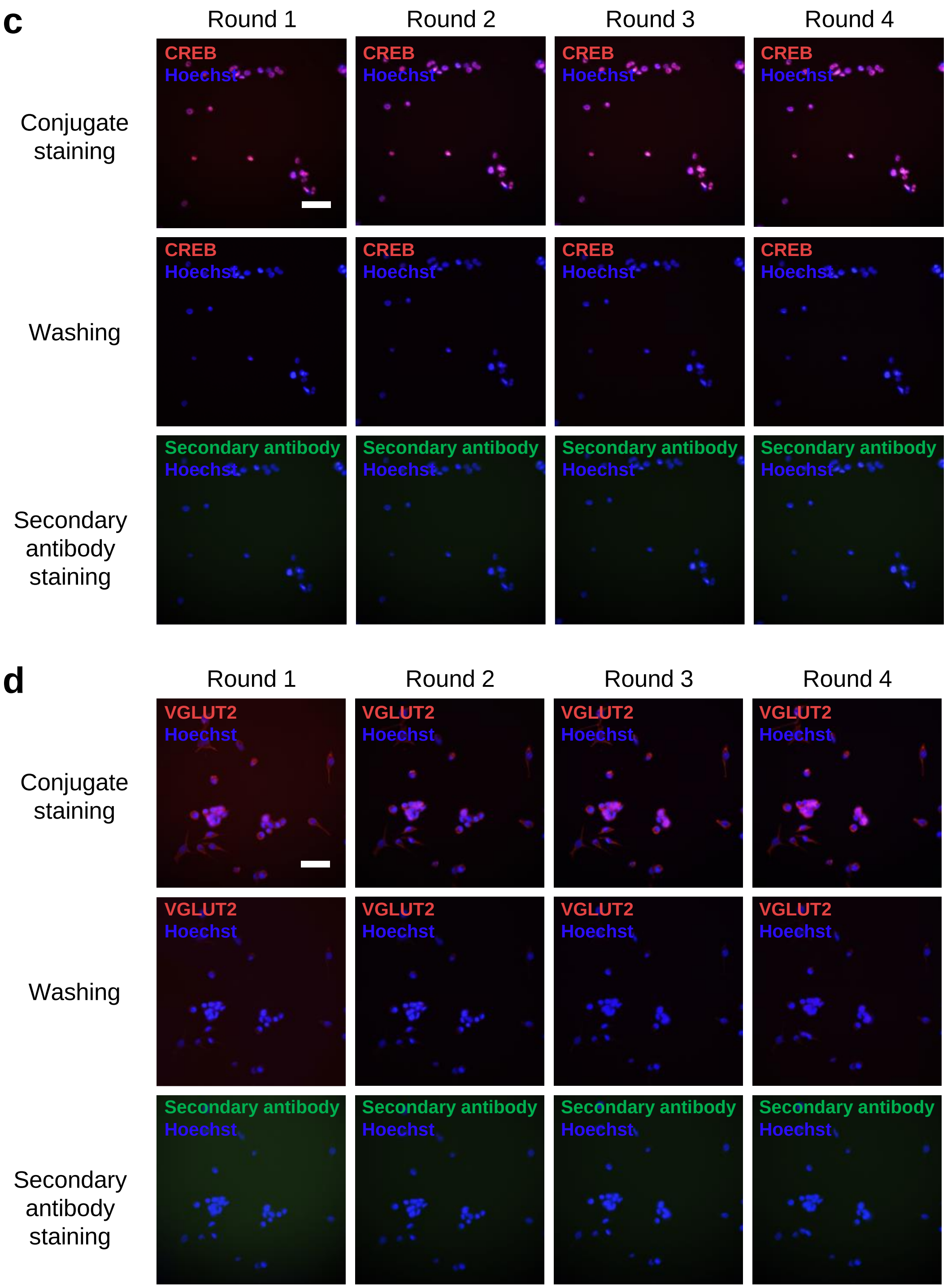
c
Round 1
Round 2
Round 3
Round 4
Conjugate staining
Washing
Secondary antibody staining
CREB
Hoechst
Secondary antibody
d
VGLUT2

Continued

e

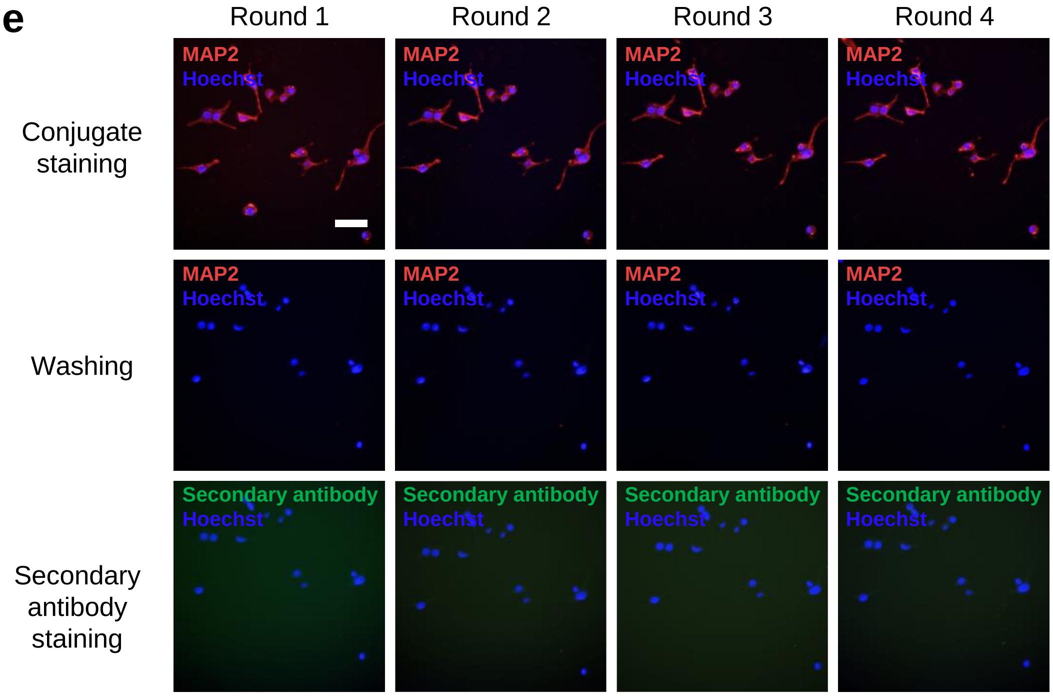

f

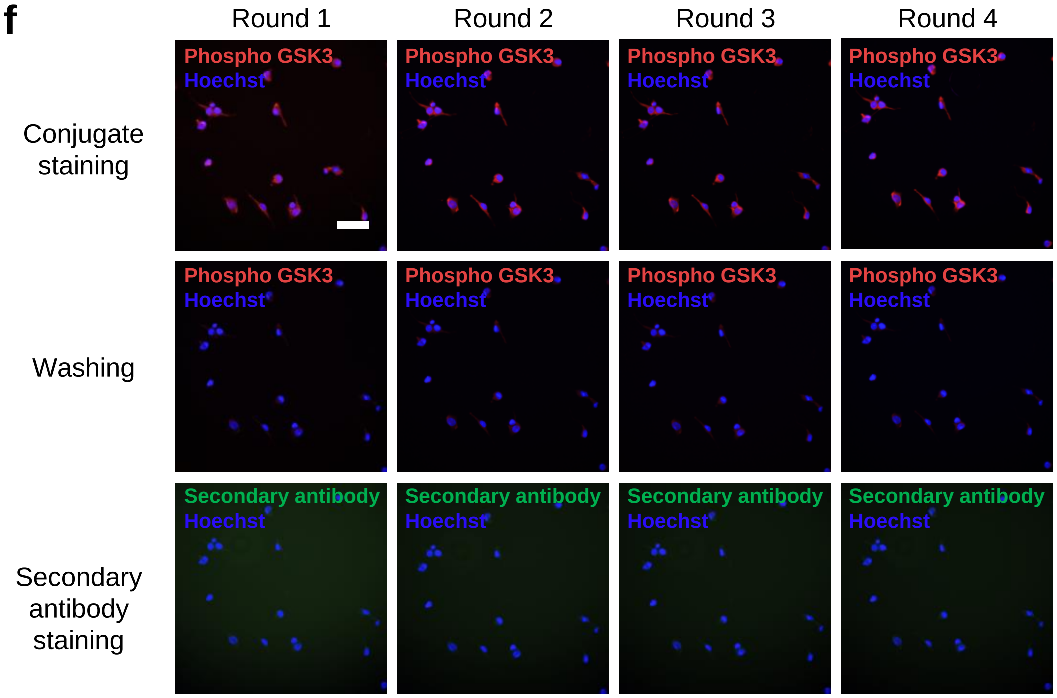

Continued

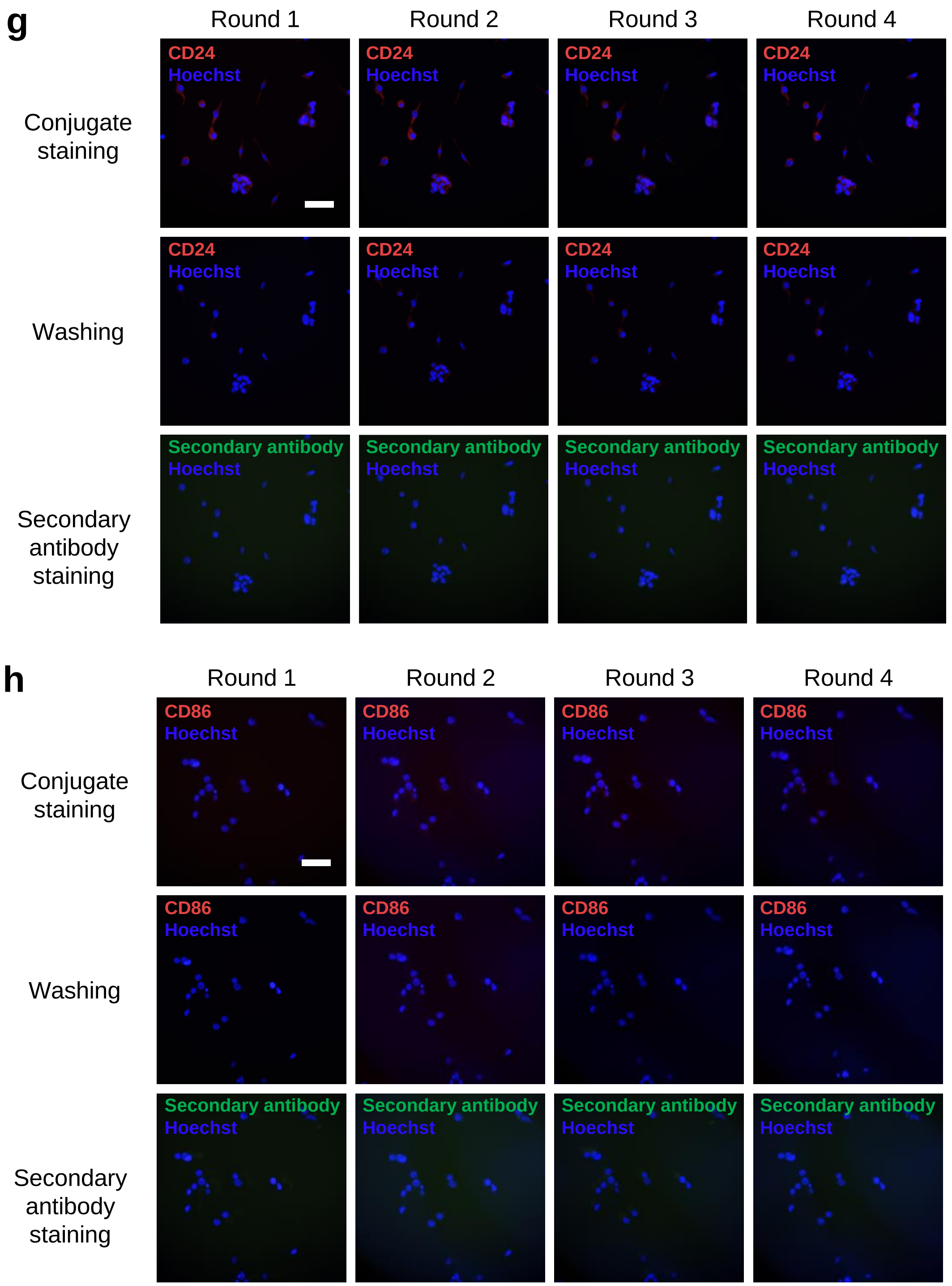

Supplementary Figure 7. Validation of conjugates staining stability and reproducibility. The differential N2a cells were treated by 4 consecutive labeling rounds with the Cy5 labeled complementary oligo- a) Tubulin, b) Lamin B1, c) CREB, d) VGLUT2, e) MAP2, f) Phospho-GSK3, g) CD24 and h) CD86 conjugates. For each labeling round, the cells were stained by Cy5 labelled conjugates, washed by a regeneration buffer, and then stained by Alexa Fluor 488 labelled secondary antibodies. Fluorescence images of the cells after each staining and washing step of the 4 consecutive labeling rounds were recorded, respectively. Scale bar = 50 μm.

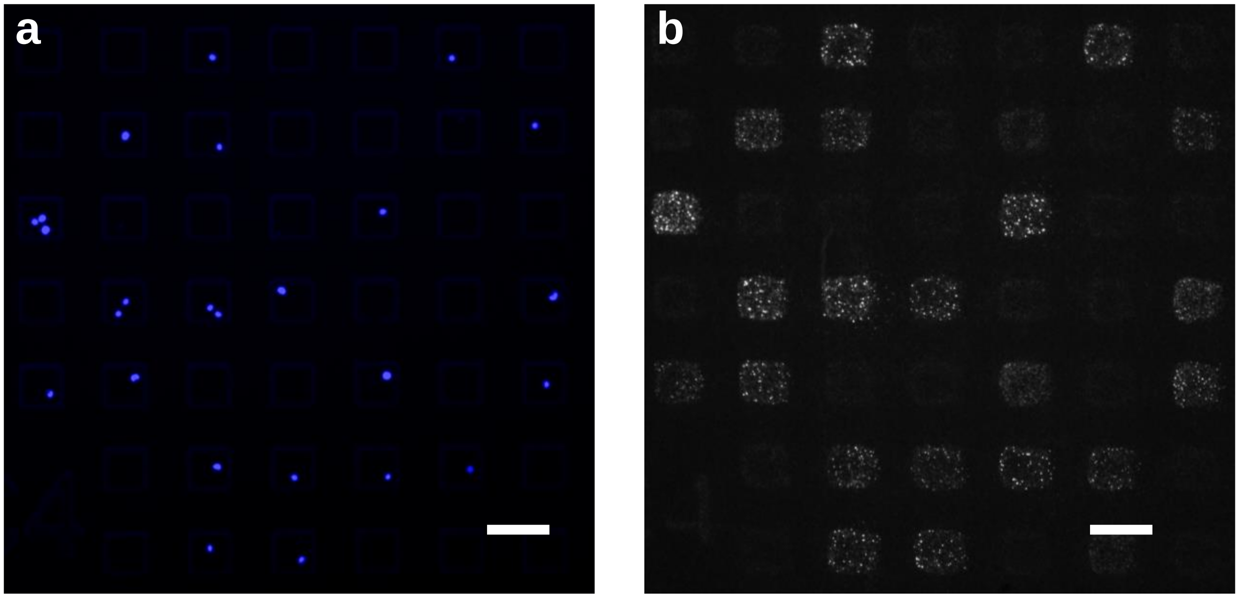

Supplementary Figure 8. Fluorescence images of a) a PDMS microwell chip containing differentiated N2a cells and b) the corresponding protein signal result on MIST array. Cells were labelled with Hoechst dye, and the protein signal was visualized by a streptavidin dye. Scale bar = 100 μm

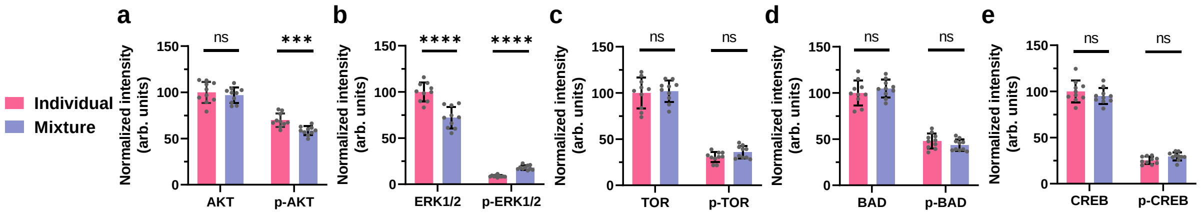

Supplementary Figure 9. Immunostaining results of differentiated N2a cells for five pairs of unphosphorylated and phosphorylated proteins: a) AKT&p-AKT; b) ERK1/2&p-ERK1/2; c) TOR&p-TOR; d) BAD&p-BAD and e) CREB&p-CREB. For the immunostaining of each pair, the cells were stained by each antibody separately and antibodies mixture together and followed by fluorophore-conjugated secondary antibodies. The fluorescence intensity was quantitatively analyzed by ImageJ and normalized by the individual staining result of unphosphorylated proteins. Data are presented as mean ± SD, n = 10 different single cells. ****p*-value < 0.001, *****p*-value < 0.0001, determined by two-tailed unpaired T test. The term (arb. units) is abbreviated for arbitrary units. The antibody parking issues are found in the staining by p-AKT, ERK1/2 and p-ERK1/2, with a positive rate of 30%.

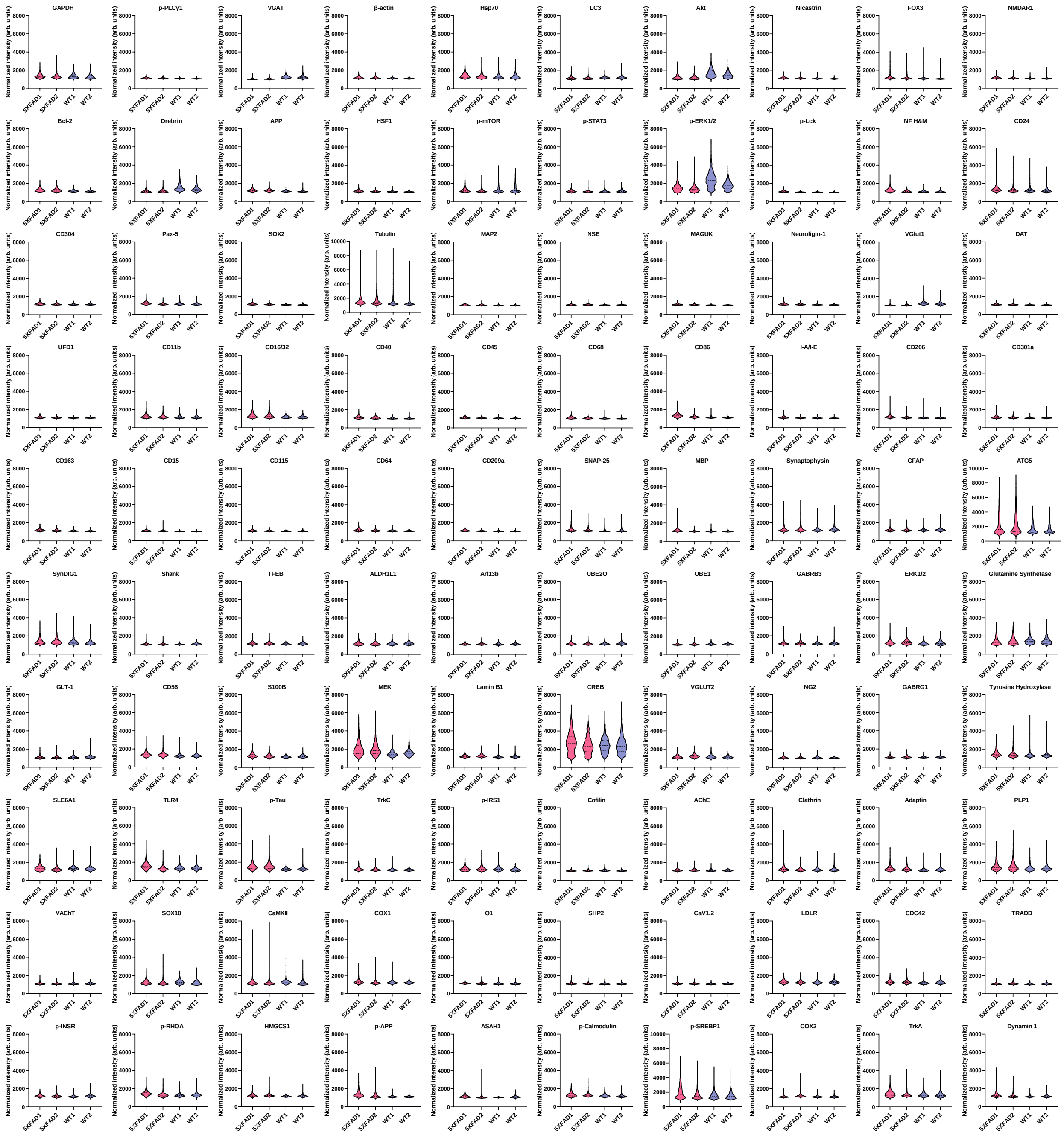
GAPDH
p-PLCγ1
VGAT
β-actin
Hsp70
LC3
Akt
Nicastrin
FOX3
NMDAR1
Bcl-2
Drebrin
APP
HSF1
p-mTOR
p-STAT3
p-ERK1/2
p-Lck
NF H&M
CD24
CD304
Pax-5
SOX2
Tubulin
MAP2
NSE
MAGUK
Neuroligin-1
VGlut1
DAT
UFD1
CD11b
CD16/32
CD40
CD45
CD68
CD86
I-A/I-E
CD206
CD301a
CD163
CD15
CD115
CD64
CD209a
SNAP-25
MBP
Synaptophysin
GFAP
ATG5
SynDIG1
Shank
TFEB
ALDH1L1
Arl13b
UBE2O
UBE1
GABRB3
ERK1/2
Glutamine Synthetase
GLT-1
CD56
S100B
MEK
Lamin B1
CREB
VGLUT2
NG2
GABRG1
Tyrosine Hydroxylase
SLC6A1
TLR4
p-Tau
TrkC
p-IRS1
Cofilin
AChE
Clathrin
Adaptin
PLP1
VAChT
SOX10
CaMKII
COX1
O1
SHP2
CaV1.2
LDLR
CDC42
TRADD
p-INSR
p-RHOA
HMGCS1
p-APP
ASAH1
p-Calmodulin
p-SREBP1
COX2
TrkA
Dynamin 1
Normalized intensity (arb. units)
5XFAD1
5XFAD2
WT1
WT2

Continued

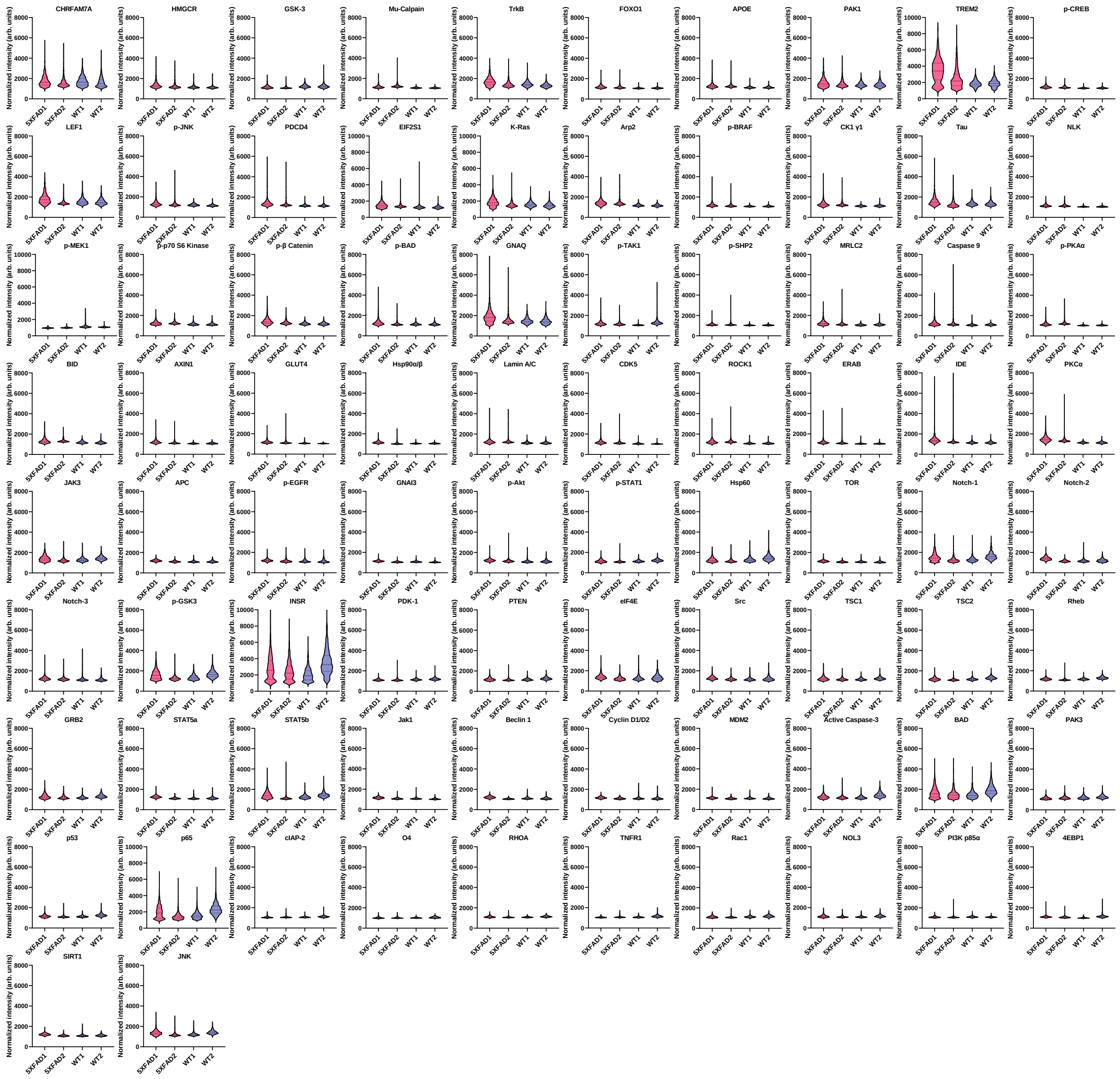

Supplementary Figure 10. Violin plots of the 182 proteins expression level measured by CycMIST on 5XFAD and WT mouse cortex samples. The term (arb. units) is abbreviated for arbitrary units.

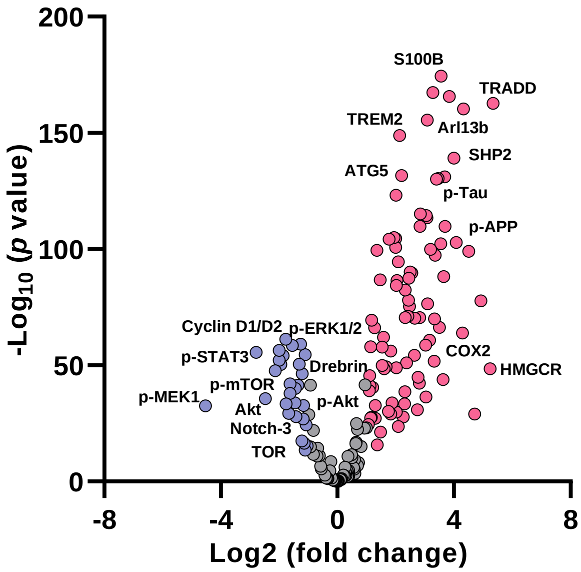

Supplementary Figure 11. Volcano plot of differentially expressed proteins in 5xFAD and WT cells. Pink dots represent proteins expressed at high levels in 5XFAD cells, and purple dots are proteins expressed at high levels in WT cells. Y axis is − log10 (*p*-values) while X axis shows $\log_2$ fold change values (two-sided *T*-test unadjusted $p \leq 0.05$ and ≥2-fold change).

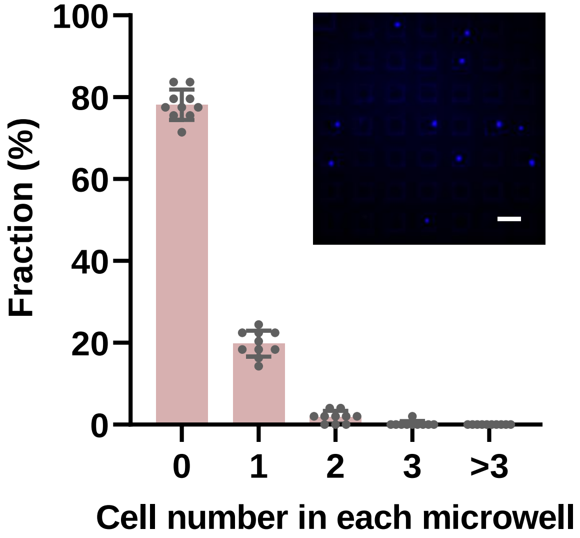

Supplementary Figure 12. The cell loading result at the cell concentration of 30,000 cells/mL for 7 mins. Inset is the fluorescence image of loaded cells into a PDMS microwell chip, in which the cells were stained by Hoechst 33342. Scale bar = 100 µm. Data are presented as mean ± standard deviations (SD), n = 10 independent experiments, error bars are within symbol size if not shown.

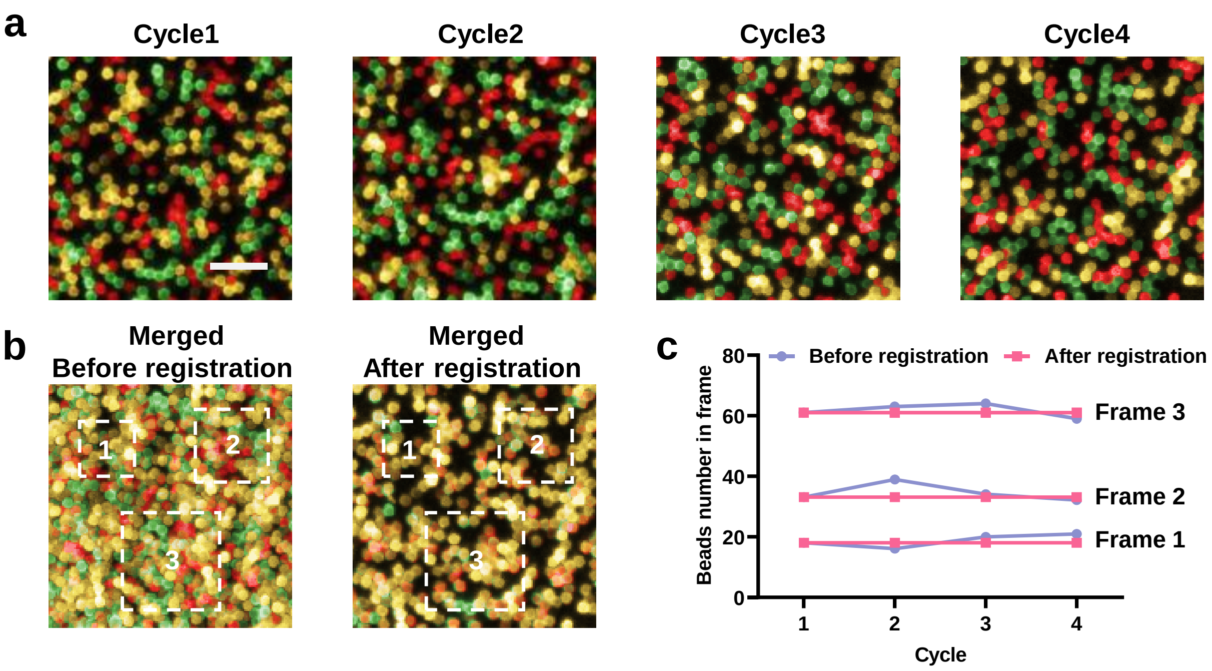

Supplementary Figure 13. a) Decoding images from four cycles. b) Merged images from the four decoding cycles before and after image registration. c) Quantification of beads number in three random areas before and after image registration. Scale bar = 10 µm for all images.
