# Supplementary material for: Cyclic microchip assay for measurement of hundreds of functional proteins in single neurons
Source: Nat Commun. 2022 Jun 21;13:3548. doi: 10.1038/s41467-022-31336-x (PMC9213506; doi:10.1038/s41467-022-31336-x)
Supplement: Supplementary file 2 — Reporting Summary [file 41467_2022_31336_MOESM2_ESM.pdf]

nature portfolio

Corresponding author(s): Jun Wang
Last updated by author(s): Jun 10, 2022

Reporting Summary

Nature Portfolio wishes to improve the reproducibility of the work that we publish. This form provides structure for consistency and transparency in reporting. For further information on Nature Portfolio policies, see our Editorial Policies and the Editorial Policy Checklist.

Statistics

For all statistical analyses, confirm that the following items are present in the figure legend, table legend, main text, or Methods section.

n/a	Confirmed	
☐	☒	The exact sample size (*n*) for each experimental group/condition, given as a discrete number and unit of measurement
☐	☒	A statement on whether measurements were taken from distinct samples or whether the same sample was measured repeatedly
☐	☒	The statistical test(s) used AND whether they are one- or two-sided<br>*Only common tests should be described solely by name; describe more complex techniques in the Methods section.*
☒	☐	A description of all covariates tested
☐	☒	A description of any assumptions or corrections, such as tests of normality and adjustment for multiple comparisons
☐	☒	A full description of the statistical parameters including central tendency (e.g. means) or other basic estimates (e.g. regression coefficient) AND variation (e.g. standard deviation) or associated estimates of uncertainty (e.g. confidence intervals)
☐	☒	For null hypothesis testing, the test statistic (e.g. *F*, *t*, *r*) with confidence intervals, effect sizes, degrees of freedom and *P* value noted<br>*Give P values as exact values whenever suitable.*
☒	☐	For Bayesian analysis, information on the choice of priors and Markov chain Monte Carlo settings
☒	☐	For hierarchical and complex designs, identification of the appropriate level for tests and full reporting of outcomes
☒	☐	Estimates of effect sizes (e.g. Cohen's *d*, Pearson's *r*), indicating how they were calculated

Our web collection on statistics for biologists contains articles on many of the points above.

Software and code

Policy information about availability of computer code

Data collection	Nikon Elements (NIS-Elements AR Microscope Imaging Software) was used to acquire images.
Data analysis	Image registration and single-cell data was analyzed using an in-house MatLab (R2020a) program code, and the code and its user instructions were uploaded as a Supplementary Software file. Fluorescence intensity of images was quantitatively analyzed by ImageJ (FIJI Version 1.53). Data plots and statistics were generated by GraphPad Prism 8.0 and Python program (3.7.9). Heatmaps and unsupervised clustering were performed with the Morpheus program (Broad Institute, Morpheus, https://software.broadinstitute.org/morpheus).

For manuscripts utilizing custom algorithms or software that are central to the research but not yet described in published literature, software must be made available to editors and reviewers. We strongly encourage code deposition in a community repository (e.g. GitHub). See the Nature Portfolio guidelines for submitting code & software for further information.

Data

Policy information about availability of data

All manuscripts must include a data availability statement. This statement should provide the following information, where applicable:

- Accession codes, unique identifiers, or web links for publicly available datasets
- A description of any restrictions on data availability
- For clinical datasets or third party data, please ensure that the statement adheres to our policy

All the data supporting the study are available within the article and its Supplementary Information files. Source data are provided with this paper.

March 2021

Field-specific reporting

Please select the one below that is the best fit for your research. If you are not sure, read the appropriate sections before making your selection.

☒ Life sciences ☐ Behavioural & social sciences ☐ Ecological, evolutionary & environmental sciences

For a reference copy of the document with all sections, see nature.com/documents/nr-reporting-summary-flat.pdf

Life sciences study design

All studies must disclose on these points even when the disclosure is negative.

Sample size	The sample size was determined based on the requirement to perform statistical tests and the availability of materials, which were indicated in the experimental sections and figure captions.
Data exclusions	No data was excluded from the study.
Replication	All data was reliable reproduced, and all measurements were performed at least three duplicates ($n \geq 3$). The exact number of repeats are provided in each figure caption and experimental sections.
Randomization	Not applicable because the work is not investigating more than two populations
Blinding	Not applicable because the work is not investigating more than two populations.

Reporting for specific materials, systems and methods

We require information from authors about some types of materials, experimental systems and methods used in many studies. Here, indicate whether each material, system or method listed is relevant to your study. If you are not sure if a list item applies to your research, read the appropriate section before selecting a response.

Materials & experimental systems

n/a	Involved in the study	
☐	☒	Antibodies
☐	☒	Eukaryotic cell lines
☒	☐	Palaeontology and archaeology
☐	☒	Animals and other organisms
☒	☐	Human research participants
☒	☐	Clinical data
☒	☐	Dual use research of concern

Methods

n/a	Involved in the study	
☒	☐	ChIP-seq
☒	☐	Flow cytometry
☒	☐	MRI-based neuroimaging

Antibodies

Antibodies used	Detailed information about antibodies used in this study (name, vendor, catalog number, isotype and clone) has been provided in Supplementary Data 2. For the primary antibody, a 50 μg of antibody at the concentration of 1 mg/mL or higher was added for the antibody-oligo conjugation. After the reaction, purification and concentration measurements, 10 μg/mL of antibody-oligo conjugate diluted in blocking buffer was used for cell staining and single-cell experiments.
Validation	Each antibody selected has been quality-tested from vendors. Moreover, all antibodies were tested on Neuro-2a cells by conventional immunofluorescence microscopy and compared with the information provided by the vendors and literature to ensure the binding and localization of each antibody with respect to its target.

Eukaryotic cell lines

Policy information about cell lines

Cell line source(s)	Neuro-2a cells (ATCC® CCL-131™).
Authentication	Cell authentication test was not performed in this study
Mycoplasma contamination	Cell lines were not tested for mycoplasma contamination but no indication of contamination was observed.
Commonly misidentified lines (See ICLAC register)	No commonly misidentified cell lines were used in this study.

Animals and other organisms

Policy information about studies involving animals; ARRIVE guidelines recommended for reporting animal research

Laboratory animals	Brains of male wild-type (WT) and C57BL/6 (5XFAD) transgenic mice with ages of 5.5-month-old were provided from four coauthors (Tanya Jain and Professor Yue-Ming Li at Memorial Sloan Kettering Cancer Center, Firoz Akhter and Professor Donghui Zhu at Stony Brook University).
Wild animals	No wild animals were used in this study.
Field-collected samples	No field-collected samples were used in this study.
Ethics oversight	The mouse work was approved by the Institutional Animal Care and Use Committee (IACUC) at Stony Brook University.

Note that full information on the approval of the study protocol must also be provided in the manuscript.
