# Supplementary material for: Cyclic microchip assay for measurement of hundreds of functional proteins in single neurons
Source: Nat Commun. 2022 Jun 21;13:3548. doi: 10.1038/s41467-022-31336-x (PMC9213506; doi:10.1038/s41467-022-31336-x)
Supplement: Supplementary file 3 — Description of Additional Supplementary Files [file 41467_2022_31336_MOESM3_ESM.pdf]

## Description of Additional Supplementary Files

**File Name:** Supplementary Data 1

**Description:** Oligo DNAs information and decoding design

**File Name:** Supplementary Data 2

**Description:** Antibody information

**File Name:** Supplementary Software

**Description:** MATLAB code and user instructions
